# Symbiont transmission in marine sponges: reproduction, development, and metamorphosis

**DOI:** 10.1186/s12915-022-01291-6

**Published:** 2022-05-06

**Authors:** Tyler J. Carrier, Manuel Maldonado, Lara Schmittmann, Lucía Pita, Thomas C. G. Bosch, Ute Hentschel

**Affiliations:** 1 GEOMAR Helmholtz Centre for Ocean Research, Kiel, Germany; 2 Zoological Institute, University of Kiel, Kiel, Germany; 3 Department of Marine Ecology, Center for Advanced Studies of Blanes (CEAB-CSIC), Girona, Spain; 4 Institute of Marine Sciences (ICM-CSIC), Barcelona, Spain

**Keywords:** Evolution, Holobiont, Host-microbe, Invertebrate, Microbiome, Porifera

## Abstract

Marine sponges (phylum Porifera) form symbioses with diverse microbial communities that can be transmitted between generations through their developmental stages. Here, we integrate embryology and microbiology to review how symbiotic microorganisms are transmitted in this early-diverging lineage. We describe that vertical transmission is widespread but not universal, that microbes are vertically transmitted during a select developmental window, and that properties of the developmental microbiome depends on whether a species is a high or low microbial abundance sponge. Reproduction, development, and symbiosis are thus deeply rooted, but why these partnerships form remains the central and elusive tenet of these developmental symbioses.

**Supplementary Information:**

The online version contains supplementary material available at 10.1186/s12915-022-01291-6.

“The ultimate goal is to characterize the mechanisms by which this fidelity is achieved and to define the impact of these associations on the evolution of developmental patterns in animals” –McFall-Ngai (2002)

## Background

Marine sponges (phylum Porifera) arose >600 million years ago and are among the oldest extant Metazoan lineages [1]. The >9300 species of marine sponges are grouped into four major classes: Calcarea (calcareous sponges; 8%), Demospongiae (demosponges; 83%), Hexactinellida (glass sponges; 7%), and Homoscleromorpha (1%) [2]. These sessile invertebrates filter-feed by pumping thousands of liters of seawater per kilogram of sponge per day, making them vital to benthic-pelagic coupling and the biogeochemical cycling of nutrients in the tropical, temperate, and polar ecosystems that they inhabit [3–5]. One of the most notable characteristics of marine sponges is how they interact with the microbial world and, particularly, in the symbioses that they form with microorganisms [6–13].

**Fig. 1:**
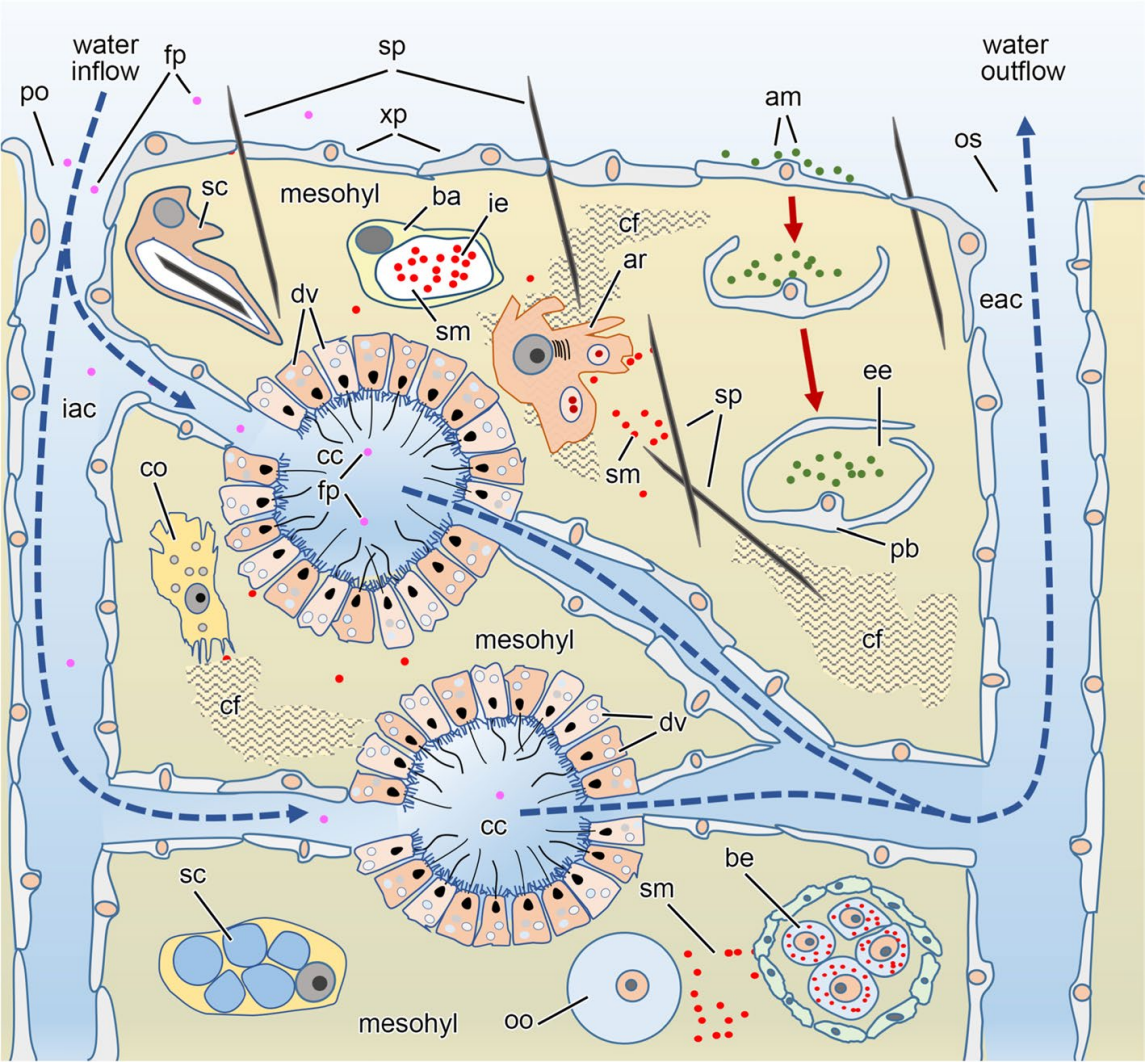
Schematic diagram summarizing the essential cytology known for demosponges, as reported previously (e.g., [18–23]). Sponges are bi-epithelial organisms. The external epithelium consists of pavement-like cells (exopinacocytes, xp). The internal epithelium delimits internal aquiferous canals and also consists of pavement-like cells (endopinacocytes, xp). However, at some points, the aquiferous canals expand into chambers, in which the pavement-like cells are replaced by cuboidal (cells provided with distal microvilli and a flagellum), the choanocytes. The beating of the choanocyte flagellum creates an inflow of ambient seawater (dashed arrows), which enters the inhalant aquiferous canals (iac) through the pores (po) at the sponge “skin,” carrying particles (fp) in suspension to the choanocyte chambers (cc). The choanocytes retain and engulf microorganisms or other particles (fp) that arrive at the lumen of the chambers, where digesting food particles into digesting vesicles (dv) begins. The seawater that is cleared of particles flows out through the exhalant aquiferous canals (eac) and the oscule (os). Between the internal and external epithelia, there is a thick region—a sort of internal “tissue”—known as the mesohyl. It consists of a gel-like intercellular medium with abundant collagen fibrils (cf) in which several types of amoeboid cells wander around. Among others, there are the collencytes (which produce the collagen fibrils, cf), sclerocytes (sc; which build the spicules, sp), and the archeocytes (which serve as cellular defense system). Archeocytes are totipotent and can transdifferentiate into almost any other cell type. In the mesohyl, there are also free-living symbiotic microbes (sm) as well as cells hosting microbes. The bacteriocytes (ba) are cells that host symbiotic microbes (sm) in the intracellular environment (ie) of a large intracytoplasmic vesicle. A peculiar type of bacteriocyte is the pocket bacteriocyte (pb). It is believed to derive from an epithelial cell that attracts free-living microbes (am) from the ambient water to the sponge surface. Then, the cell leaves the epithelium to enter the mesohyl (red arrows), folding over itself to form an extracellular sack-like cavity, in which microbes are host in the extracellular environment (ee). Oocytes (oo) and brooded embryos (be) also develop in the mesohyl

Sponges can interact with trillions of microbes each day by filter-feeding alone [5, 14], and they use an intricate set of morphological, cellular, and molecular dialogs to distinguish between friend, foe, and food [7, 15–17]. Microorganisms suspended in the seawater flow through the pores and inhalant aquiferous canals to the choanocyte chambers (Fig. 1). A fraction of these microbes are able to elude digestion and migrate through the epithelia of the aquiferous canal or the choanocyte chambers. These microbes will ultimately enter the mesohyl, where they can proliferate and provide a functional contribution to the sponge holobiont [6, 16, 24, 25]. In the sponge mesohyl (Fig. 1), there is typically an assemblage of microorganisms (~10^6^ to ~10^9^ cells per mL) that, in some species, can equate to ~40% of sponge tissue by volume [26]. Symbionts, defined as “dissimilar organisms living closely together in a sustained interaction” [27, 28], of sponges are taxonomically and functionally diverse. Sponges harbor 63 bacterial phyla (e.g., Proteobacteria, Actinobacteria, and Chloroflexi) that are often metabolically intertwined with the host through the utilization of dissolved organic and inorganic carbon and nitrogen as well as through amino acid and vitamin biosynthesis [6, 7, 9, 11, 29–31]. In addition to the bacterial symbionts, the microbial communities associated with sponges also includes archaea, viruses, and unicellular eukaryotes (e.g., *Symbiodinium*, other dinoflagellates, and diatoms) [9, 18].

Properties of these symbiont communities depend on whether a species is a “low microbial abundance” (LMA) or “high microbial abundance” (HMA) sponge. LMA sponges harbor microbial populations that are roughly equal to the microbial densities of the surrounding seawater while those of HMA sponges—formerly called bacteriosponges—are two to four orders of magnitude higher than LMA sponges [7, 14, 32, 33]. These two patterns of microbial abundance have evolved independently on numerous occasions and have resulted in fundamental differences in the taxonomic composition and function of sponge symbiont communities [32, 33]. HMA sponges are comparably enriched in Acidobacteria, Chloroflexi, and Poribacteria, while LMA sponges are comparably enriched in Cyanobacteria and Proteobacteria [31, 33]. Moreover, symbionts of HMA sponges are primarily responsible for autotrophic and heterotrophic metabolism while these processes are primarily carried out by the host in LMA sponges [34, 35]. The provisioning of these nutritional resources from parent to offspring are then essential for reproduction [36–38].

**Fig. 2:**
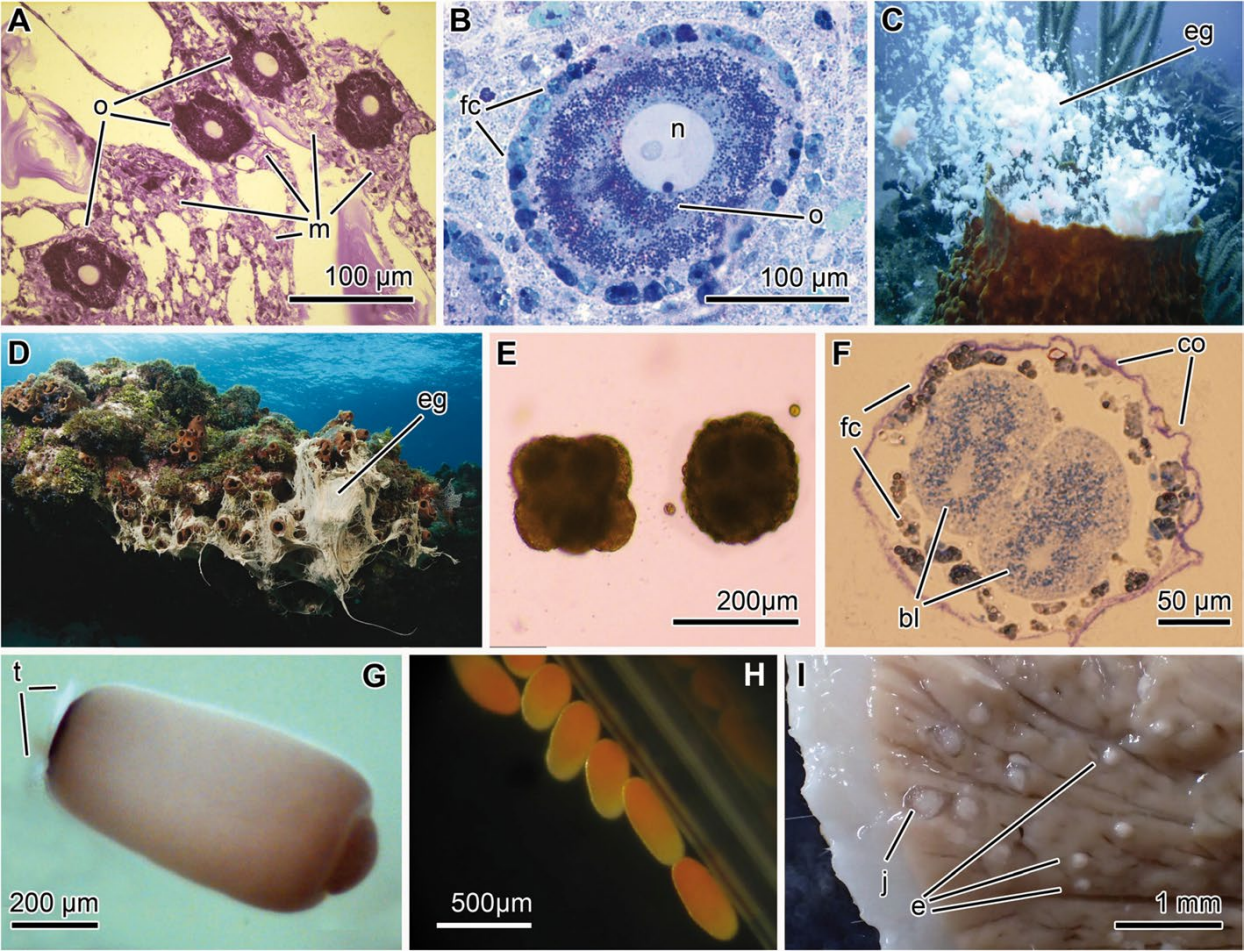
Summary of the main events in sponge reproduction and development. **A** Amoeboid oocytes (o) in the mesohyl (m) of the demosponge *Agelas oroides* collected in August 2018 and first described here*.* **B** Late-stage oocyte with nucleolated nucleous surrounded by a layer of folicular cells (fc) within the mesohyl of the demosponge *Aplysina aerophoba* [43]. **C** Release of egg (eg) masses by the demosponge *Xestospongia muta* (unpublished, UH). **D** Release of eggs (eg) in mucous strands by the demosponge *Agelas sceptrum* (photo by Stephen Frink)*.* **E** Morules of *A. aerophoba* developing externally in the water column [43]. **F** Section of a morula of *A. aerophoba* similar to those in Fig. 1E, showing that they are surrounded by a collagen envelop (co) and a layer of follicle cells (fc). **G** A free-swimming parenchymella larva of the demosponge *Ircinia felix*, showing a posterior tuft of long cilia, which are part of a larval photoreceptor organ [44]. **H** Non-tufted parenchymella of the demosponge *Ectyoplasia ferox*, with a translucent posterior pole [45]. **I** Hand-made section through the body of an individual of demosponge *Craniella zetlandica* that was collected from a reef of the cold-water coral *Lophelia pertusa* in the Stjersundet area of a Norwegian fjord in August 2018 (unpublished, MM). This species undergoes a direct development and, therefore, lacks the larval stage. The picture shows smaller embryos (e) being brooded in deepest regions of the body, which migrate towards the surface of the sponge during development to be finally released as miniature sponge (juvenile = j) by squeezing themselves through the cells and spicules of the sponge cortex

Most marine invertebrates, including sponges, exhibit a biphasic (indirect) life cycle, where adults reside on the seafloor and the developmental stages are suspended in the water column [39–42]. Sponges predominately fertilize internally, brood their embryos, and then release swimming larvae (viviparity). Fewer species broadcast spawn their gametes for external development (oviparity), while others develop directly by bypassing the larval stage and having juveniles crawl out from the parent (Fig. 2). Demosponges can either be viviparous or oviparous while Homosclerophorida and Calcarea are viviparous [46, 47]. The reproductive tendencies of Hexactinellida remain largely uninvestigated, but the few reported cases show that embryos are brooded [48, 49], which should lead to viviparism. Once the larva forms internally or externally, it undergoes a lecithotrophic (non-feeding) development that generally lasts a few hours to several days, during which the larva may disperse a few meters to hundreds of kilometers [41, 50–54]. The developmental stages of sponges also serve as the predominant vector of symbiont transmission between generations.

Symbioses can be maintained with a high fidelity through both horizontal and vertical transmission [55–58]. Horizontal transmission occurs when a symbiosis is non-continuous throughout the host life cycle and resumes following an environmental acquisition [55, 57]. One example is the mutualism between the Hawaiian bobtail squid *Euprymna scolopes* and the luminous bacterium *Vibrio fischeri*. In this partnership, embryonic squid lack symbionts until post-hatching juveniles recruit *V. fischeri* from the seawater [59–61]. Vertical transmission, on the other hand, are symbioses that are maintained throughout the host’s life cycle and across generations [55, 57]. This type of transmission is well-studied in aphids, where cells of the nutritional mutualist *Buchnera* sp. are endocytosed at the posterior pole of mature eggs [62]. Both transmission modes are ancient, evolutionarily advantageous, and not mutually exclusive [10, 13, 55, 57, 63, 64]. Horizontal and vertical transmission both occur in sponges and enable their symbiotic lifestyles to be maintained.

Researchers in the 1930s first realized that microorganisms were present in the tissues of marine sponges [65]. These microbes were initially regarded as pathogens, but were reinterpreted as probable mutualists once microbes were consistently observed in abundance across sponge species [66]. The transmission of microorganisms in marine sponges was not identified until the 1960s when Lévi and Porte [67] used transmission electron microscopy to describe the cellular structures of *Oscarella lobularis* larvae. Symbiont transmission has since become a fundamental subdiscipline of sponge microbiology, and this manuscript is an effort to synthesize ~60 years of research on the diversity and intricacies of symbiont transmission. We will qualitatively address three main questions. First, is symbiont transmission universal in this early-diverging lineage and what are the underlying mechanisms? Second, are patterns of microbial abundance in the adults reflected in the diversity and composition of the transmitted symbionts? Lastly, does symbiont function influence the larvae while in the pelagic environment? We conclude by providing a framework to assess the functional mechanisms of developmental symbioses in this emerging experimental system.

## Paths and patterns of symbiont transmission

Vertical transmission requires coordination between the developmental programs of the host and the microorganisms [57]. To understand how symbionts are maintained between generations, we must first briefly address how gametes arise (gametogenesis) and how they develop (embryogenesis). This baseline will set the stage to detail the morphogenetic events involved in symbiont transmission during reproduction and development. We then describe the transmission of microbes from parent to offspring, including the types of microbes and the developmental stages transmittal occurs. We conclude this section with a meta-analysis of the bacterial communities associated with the developmental stages of marine sponges.

### Reproduction and development

Marine sponges can either be separate sexes (gonochoristic) or both sexes occur simultaneously in an individual (hermaphroditic). Gametoblasts (i.e., mother cells of gametes) are often motile and capable of actively migrating towards their nutrition source [68, 69]. Gametogenesis is initiated when the expression of genes relating to cell proliferation and pluripotency induce choanocytes and pluripotent archaeocytes to transdifferentiate into oogonia and spermatogonia [46, 70]. Established oogonia undergo meiosis, with the subsequent oocytes maturing during vitellogenesis as the oocyte becomes enriched with yolk [68, 69]. Spermatogonia reach maturation through two rounds of meiosis that greatly reduce cell volume and cytoplasm content. A flagellum is then built to become a mature spermatozoon [46, 70].

Mature spermatozoa are released through the excurrent flow of males (or hermaphroditic sponges serving as functional males), and mature oocytes may be fertilized in one of two ways. First, spermatozoa of broadcast spawning (oviparous) sponge species may encounter a conspecific oocyte that was released into the water column [71]. Second, gravid females (or hermaphrodite individuals serving as functional females) of internally developing species (viviparous) may capture conspecific spermatozoa in their inhalant flow [72, 73]. These spermatozoa are transferred to choanocyte chambers that phagocytose, but do not digest, the engulfed spermatozoa through unknown molecular signaling processes (e.g., [74]). The choanocytes with spermatozoa then leave the epithelium and become amoeboid cells that wander through the mesohyl seeking a mature oocyte. Each spermatozoon is carried individually within a cytoplasmic vesicle (spermiocyst) that transfers the spematozoon nucleus directly to an oocyte for fertilization. This internal fertilization typically—but not always—leads to the subsequent embryo being brooded in the mesohyl until the larva is released from the parental sponge [50].

Embryogenesis in these early-diverging metazoans is complex and is best studied in species that develop internally [51, 72, 75, 76]. Depending on the sponge lineage, cleavage is holoblastic, equal or unequal, and may either be synchronous or asynchronous. Embryogenesis takes between a few hours to a couple days in externally developing species (Fig. 1E, F [43, 46, 77]) to a couple weeks to months in brooding species [75]. Embryos typically develop into one of eight larval types recognized to date: the amphiblastula, calciblastula, cinctoblastula, clavablastula, dispherula, hoplitomella, parenchymella, or trichimella [51, 75]. Alternatively, some sponge species (e.g., sphirophorid and stylocordylid demosponges) lack this all together by developing directly into juveniles that crawls out of the parental body [78–80].

### Symbiont transmission during reproduction and development

**Fig. 3:**
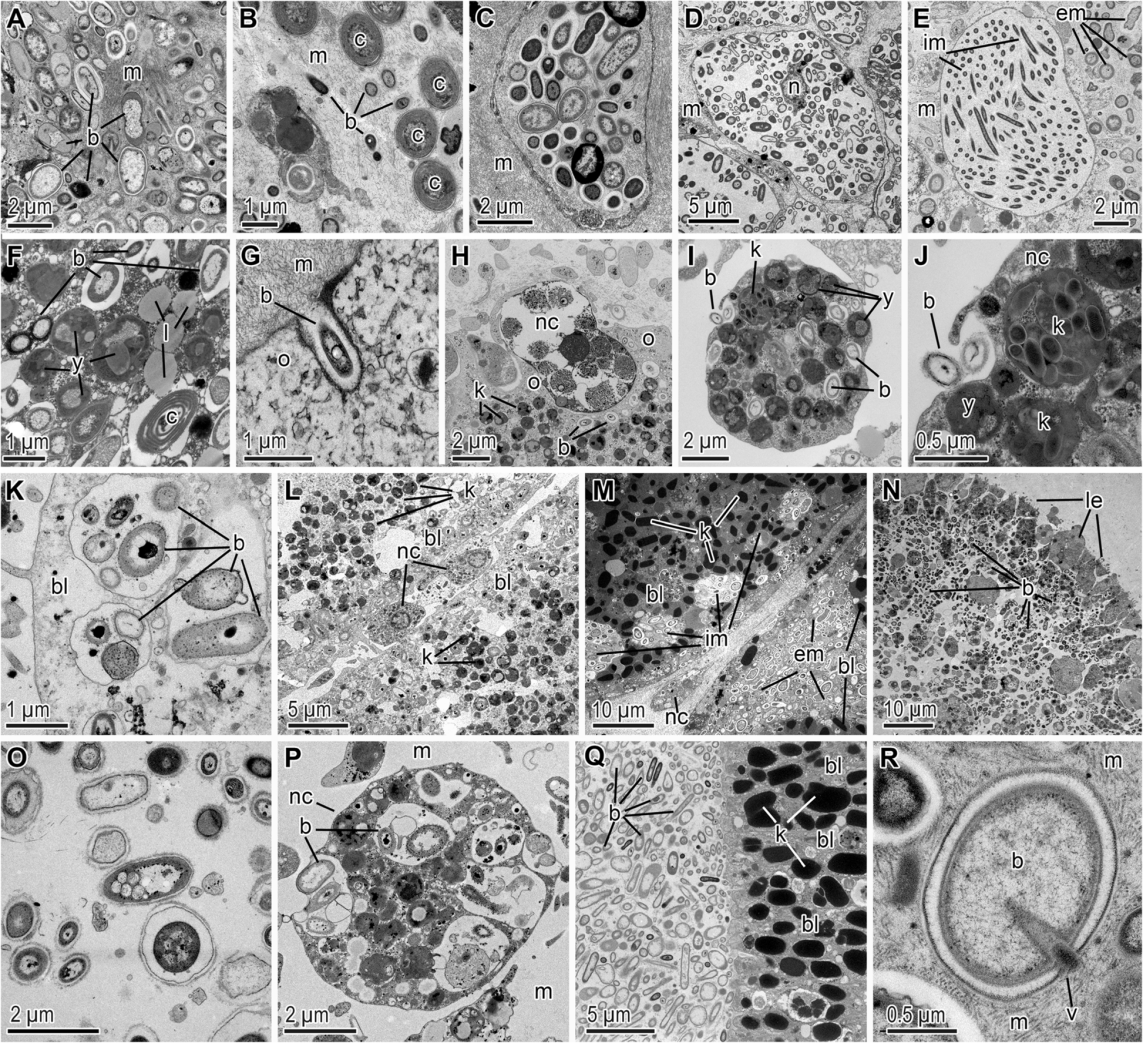
Transmission electron micrographs documenting the variety of microbes in sponges and the main mechanisms of transference. **A–C** Diversity of microbes in an undescribed *Thymosia* sp. (an HMA demosponge), where bacteria+archaea (**A**) as well as and cyanobacteria (**B**) freely co-exist in the mesohyl and with microbial population in bacteryocites (**C**) [81]. **D, E** Mesohyl of HMA demosponges *Petrosia ficiformis* (**D**) and *Aplysina cavernicola* (**E**). The former shows a pocket bacteriocyte that has a mix of microbes while the latter is a regular bacteriocyte with a single strain of a Spirochaete-like bacteria [73, 81]. **F** Cytoplasm of a *Chondrilla caribbea* (HMA demosponge) oocyte filled with bacteria+archaea, cyanobacteria, yeasts, and lipid bodies [82]. **G** Young oocyte of *Corticium candelabrum* (HMA homosclerophorid) phagocytosing a bacterium from the mesohyl [74]. **H** Oocyte of *Aplysina aerophoba* (HMA demosponge) phagocytosing a nurse cell. **I,J** Nurse cell of *C. caribbea* charged with bacteria+archaea, yeast, and yolk precursors. Note that the nurse cell is also in the process of phagocytosing two bacteria (**J**). **K, L** Morula of *A. aerophoba* that has incorporated bacteria+archaea into the blastomeres (**K**) and with nurse cells being incorporated by immigration through the cleavage furrows (**M**). Morula of *C. candelabrum* in which both free-living microbes and nurse cells are migrating through the cleavage furrows between the blastomeres. Note that blastomeres have also phagocytosed groups of bacteria to make them intracellular. **N–P** Swimming larva of *A. erophoba* that has accumulated bacteria+archaea below the larval epithelium (**N**) and making of a diverse microbial array (**O**), which is also phagocytosed and used as a food source by amoeboid cells in the larval interior (**Q**). Swimming larva of *C. candelabrum*, showing a dense a diverse assemblage of bacteria+archaea within its internal, central cavity. **R** Coccoid bacterium in the mesohyl of *Thymosia* sp. nov being infected by a bacteriophage virus. Labels: “b”= bacteria+archaea, “c”= cyanobacterium, “y”= yeast, “v” virus, “im”= intracellular microbes, “em”= extracellular microbes, “m”= mesohyl, “ns”= nurse cell, “o”= oocyte, “n”= cell nucleus, “l”= lipid body, “k”= yolk bodies, “bl”= blastomere, “le”= larval epithelium. All samples studied were collected and pictured by MM

**Fig. 4:**
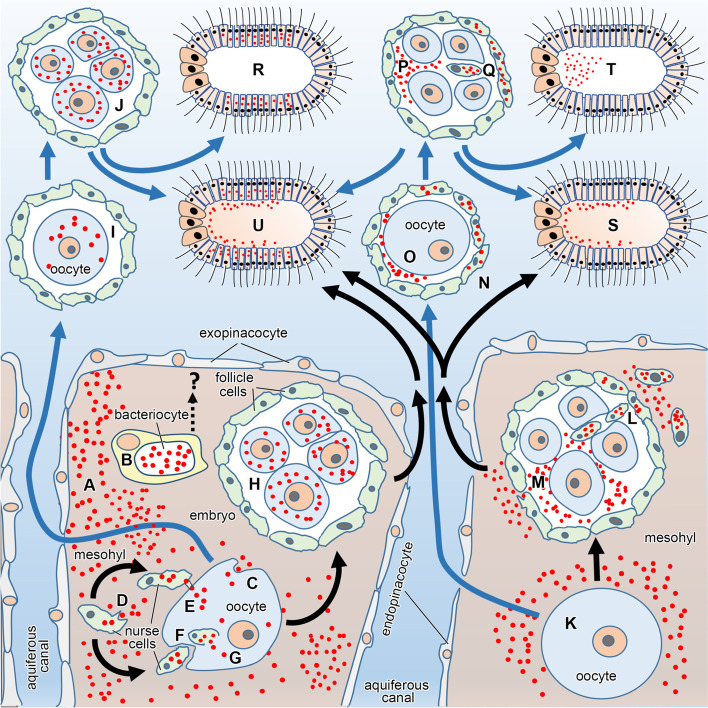
Comprehensive schematic summarizing vertical transmission of microorganisms in sponges, as reported previously [18, 19, 46, 75, 83]. Microbes (red dots) occur freely in the sponge mesohyl (A) or within bacteriocytes (B). While it is not known how microbes in bacteriocytes are transferred (?), microbes from the mesohyl are transmitted from mother to offspring through a variety of pathways (C–Q), irrespective of whether oocytes and embryos are brooded (black arrows) or develop externally (blue arrows). Oocytes can acquire microbes by direct engulfing them (C) or through nurse cells. Nurse cells engulf microbes from the mesohyl (D), which are subsequently transferred to the oocyte through cytoplasmic bridges (E) or by nurse cells being phagocytosed by the oocyte (F). Oocytes charged with microbes give rise to embryos that have intracellular microbes in the blastomeres, irrespective of whether the embryo was brooded (H) or was released into the environment (I) for external fertilization and development (J). Alternatively, oocytes may not acquire microbes (K) but an embryo (morula) can acquire them through two pathways. First, follicle cells and/or nurse cells charged with microbe that enter the embryo by migrating through the cleavage furrows (L–Q). Second, microbes infiltrated in the space between the follicle and the embryo migrating themselves through the spaces between blastomeres (M–P). These two extracellular transfers of microbes to embryos occur both in brooded embryos (L–M) and externally fertilizing oocytes (N) leading to externally developing embryos (P–Q). Therefore, the resulting larvae may have microbes in their epithelial cells (R), their internal extracellular medium (S), or their internal cavity (T), if any. By combining mechanisms for intracellular and extracellular transference, some species have larvae containing intracellular and extracellular vertically transmitted microbes (U)

Microbes within the sponge mesohyl are either free-living (Figs. 3A, B and 4A) or within specialized vesicles (bacteriocytes) (Figs. 1, 3C–E, and 4B) [18, 84, 85]. Symbionts living in the intercellular medium of the mesohyl are transferred from adult to offspring that are brooded (black arrows in Fig. 4) or that develop externally (blue arrows in Fig. 4). Pre-vitellogenic oocytes, which are ameboid cells moving through the mesohyl, can actively engulf microbes with their pseudopodia (Fig. 3G), but these microbes are thought to serve as a nutritional resource for the young oocyte [18, 86]. Just prior to vitellogenesis, the zone directly surrounding the oocyte becomes densely populated with microbes, which can be incorporated into the oocyte at the onset of vitellogenesis [18, 44, 87–89]. It is unclear whether these microbial aggregations are the result of migration or enhanced rates of proliferation around the oocyte. While the mechanisms involved are unknown, it seems likely that this process is stimulated by chemical cues from the oocyte.

Symbionts that are transmitted during vitellogenesis reach the cytoplasm of the oocyte through two primary processes. First, microbial cells are recognized at the cell surface of the oocyte and the expression of genes corresponding with vesicle transport, endocytosis, and phagocytosis—and the subsequent production of those proteins—signal for these cells to be engulfed (Fig. 3G) [70]. Second, microbial cells in the mesohyl are taken up by nurse cells (Figs. 3I, J and 4D), the cell type that is primarily responsible for provisioning materials to the oocyte [68, 69]. Nurse cells then migrate to the vicinity of the oocyte’s membrane, and microbes can be transferred through transient cytoplasmic bridges (Fig. 4E) [18, 87, 88, 90, 91]. Alternatively, entire nurse cells can be engulfed by the oocyte and its symbiont-containing vesicles are exocytosed into the cytoplasm of the oocyte (Figs. 3H and 4F, G). Near the end of vitellogenesis, nurse cells are organized into an epithelium (the follicle) that closely surrounds the oocyte (Fig. 2B and 4). This structure forms in species where the oocyte develops externally or is brooded (Fig. 4).

Once the oocyte is fertilized, the zygote begins to cleave and the multicellular embryo becomes a morula (Fig. 4G, H, K–M for internal development and Fig. 4I, J for external development). If symbionts had previously been transmitted to the cytoplasm of the oocytes (Fig. 4C–G) then those symbionts are transferred to the blastomeres of the morula (Fig. 4H, J). If symbionts had not previously been transmitted (Fig. 3K for internal development and Fig. 4N for external development), then there are two primary pathways for a morula to obtain parental symbionts. First, symbionts may be incorporated by nurse cells and/or follicle cells (Fig. 3L for internal development and Fig. 4N, Q for external development), which subsequently migrate into the morula through the spaces between the blastomeres [43, 51, 92, 93]. Symbionts of nurse cells may also be transmitted to larvae that develop externally (Fig. 4N–Q), where nurse cells degrade while the embryo is in the water column and allow for microbes to be released into the blastocoel [93]. Second, a morula may acquire symbionts when microbial cells enter the space between the follicle and the oocyte (Fig. 4M for internal development and Fig. 4O–P for external development). Symbionts may then proliferate throughout early development and then enter the morula through spaces between the blastomeres [18, 94]. To our knowledge, it remains unknown: (i) whether embryos and larvae have bacteriocytes or (ii) if bacteriocytes are also transferred through cleavage furrows. These are herein hypothesized as a plausible pathway to also transmit symbionts across sponge generations.

As result of the processes described above, early-stage larvae are transmitted a combination of extracellular (Fig. 4S–U) and intracellular (Fig. 4R) microbes. Solid larvae (e.g., parenchymella, trichimella, hoplitomella) (Fig. 4S, U) typically have extracellular symbionts in the intercellular medium underneath the larval epithelium (Fig. 3N–P). Hollow larvae (e.g., amphiblastula, calciblastula, cinctoblastula, clavablastula) predominately have symbionts in the larval cavity (Figs. 3Q and 4T), irrespective of whether this is a blastocoel or a secondary cavity [18, 90, 95–98]. During subsequent morphogenic events, intracellular symbionts remain mostly in the cell types derived from the larval epithelium while the extracellular microbial populations grow as the central cavity of the embryo expands throughout development (Table S1) [18, 75, 90, 95, 98–101].

### Global patterns in vertical transmission

We utilized the literature on sponge reproduction and development to summarize the patterns of symbiont transmission from parent to offspring in this early-diverging lineage (see, Table S1). Vertical transmission of microbial symbionts has been documented in most, but not all, of the sponge species where it has been studied (Table S1). Three of these species (*Halisarca dujardini*, *Mycale laxissima*, and *Petrosia ficiformis*) have also been reported not to vertically transmit microbial symbionts [73, 102–104]. These sponge species have been sampled globally but come predominately from the Mediterranean Sea, Northwestern Atlantic Ocean, and Caribbean Sea. A few species have also been studied in the Coral Sea/Great Barrier Reef, Scotia Sea, and Celtic Sea. Moreover, all but two species are from coastal waters, with *Craniella zetlandica* and *C. infrequens* being from deep waters in the North Atlantic Ocean. Thus, there is a strong taxonomic, geographical, and habitat bias that may confound our understanding of the major patterns of symbiont transmission in sponges.

Marine sponges vertically transmit multiple types of microorganisms, which includes archaea, bacteria, and eukaryotes. Bacteria were vertically transmitted in most, but not all, of sponge species known to inherited microbes while few species inherited archaea and eukaryotes (i.e., *Symbiodinium* spp.) (Table S1). Different types of microorganisms can be vertically transmitted simultaneously. Several sponge species inherit both archaea and bacteria, while *Chondrilla nucula* vertically transmits bacteria and yeast (Figs. 2F and 4B; Table S1) [18, 82, 105]. Interestingly, no studies have noted whether sponges vertically transmit viruses (Fig. 3R), despite their extraordinary diversity, functional relevance, and ecological importance to the adults [106–110]. The methodology for profiling, annotating, and imaging sponge viruses has developed significantly in recent years [111, 112], and it may now be methodologically feasible to determine whether sponges also vertically transmit viruses. Moreover, it remains unknown whether bacteria are the primary type of microbe that is vertically transmitted or whether this is due to a methodological bias.

The majority of sponges brood their offspring until they are early-stage larvae and microbial symbionts can, in principle, be incorporated at any time during this developmental window. Microorganisms have been detected in each of the three major developmental stages (oocytes, embryos, and larvae), with detection in larvae seemingly twice as common as in oocytes or embryos (Table S1). This distribution, however, is suspected to be confounded by a methodological bias. Studies supporting that vertical transmission occurs in oocytes and embryos predominately use electron microscopy (e.g., [18, 94]) while those suggesting the same for larvae use next-generation sequencing (e.g., [113, 114]). Therefore, we suspect that this distribution is more skewed towards oocytes and embryos than is currently represented.

Symbionts may also enter sponge sperm (Table S1). About 1.6% of *Chondrilla australiensis* spermatozoa harbor cyanobacteria, and these microbes actively divide in the sperm head [91]. It remains unknown whether a fitness benefit—possibly through a metabolic contribution—provided by these cyanobacteria would compensate for the inconvenience of the increased mass during the race to fertilize an oocyte [115]. Moreover, spermatozoa in the few congeneric species that have been examined by transmission electron microscopy lack cyanobacteria [43]. Vertical transmission through spermatozoa appears to be the exception and not the rule in marine sponges.

### Bacterial communities of the developmental stages

**Fig. 5:**
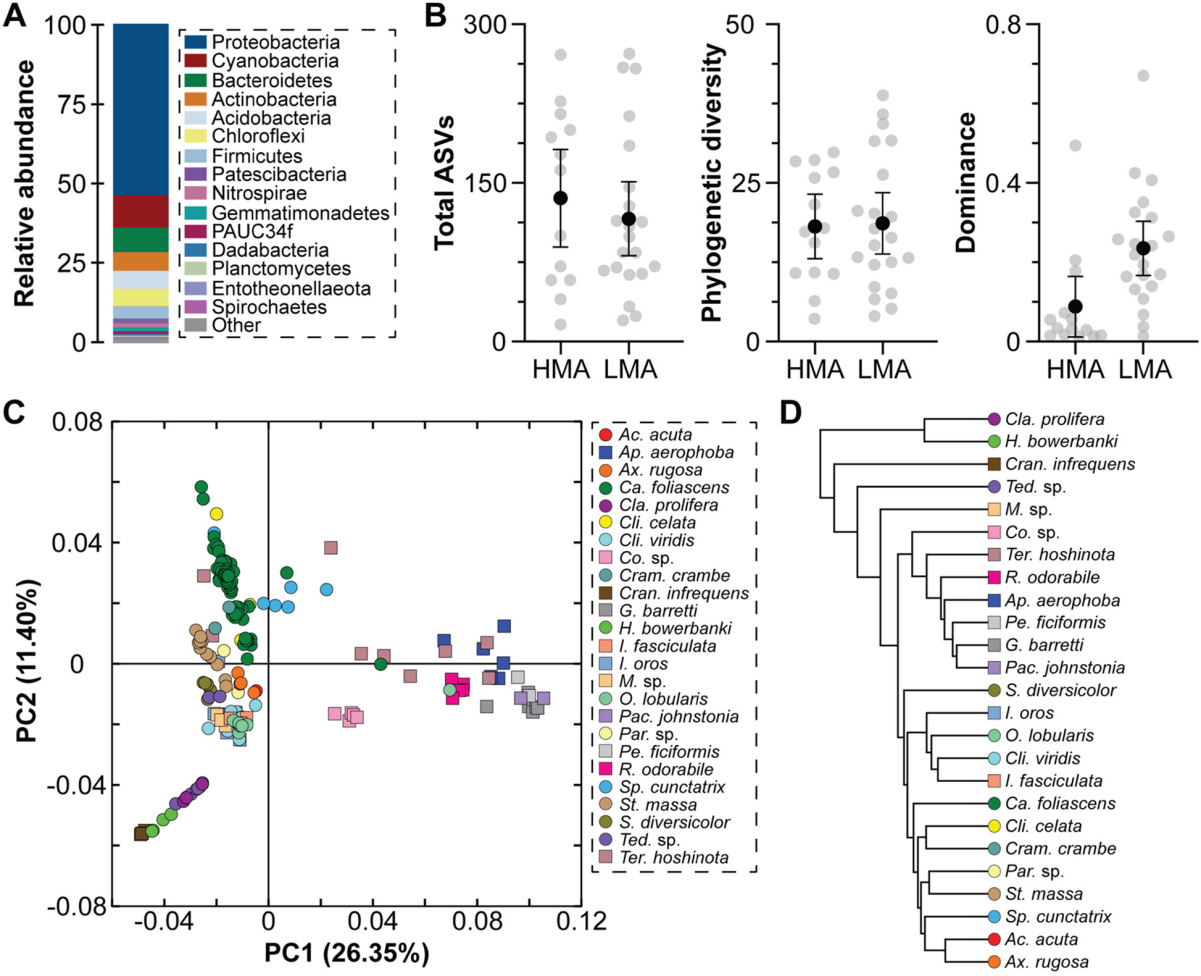
Bacterial communities of sponge developmental stages. **A** Average phyla-level taxonomic profile for the developmental stages of 35 sponge species (33 Demospongiae and 2 Homoscleromorpha; 14 HMA species and 21 LMA species) [96, 114, 116, 117]. This community includes 7794 unique taxa (ASVs) from 40 phyla and were predominantly composed of Proteobacteria (56.3%), Chloroflexi (9.8%), Acidobacteria (7.6%), Actinobacteria (5.2%), Cyanobacteria (4.0%), and Firmicutes (3.6%). **B** Diversity of the bacterial communities associated with sponge developmental stages was assessed by calculating the total ASVs, Faith’s phylogenetic diversity, and McIntosh dominance. The gray dots represent an average value for each of the 35 species and the black dot is the average for all species (± 95% confidence intervals). HMA and LMA sponges associated with bacterial communities that were comparably similar in total taxa and phylogenetic diversity and LMA sponges were dominated by a few bacterial taxa and HMA sponges were more evenly distributed. **C** Principal coordinates analysis depicting community relatedness of microbiome composition (based on weighted UniFrac values) and show that the developmental stages of sponges tend to associate with a species-specific microbiota while HMA and LMA sponges associate bacterial communities that are compositionally distinct, providing the first demonstration of the HMA-LMA dichotomy in the developmental stages. **D** Dendrogram of microbiome relatedness for the 25 sponge species with two or more samples, as based on and simplified from the principal coordinates analysis (**C**). Each sample in **C** and **D** is represented as a dot. All species are color coded and sponge lifestyle is coded by shape (HMA sponges are represented by squared and LMA sponges are represented by circles). To analyze these amplicon data, raw reads (along with quality information) from four studies [96, 114, 116, 117] were downloaded directly from the Sequence Read Archive of the National Center for Biotechnology Information (Table S2) and were imported into QIIME 2 (v.2019.1 [118]). Primers were trimmed, sequences were filtered by quality score, and each sample was denoised using Deblur [119]. QIIME 2-generated “features” were analyzed as amplicon sequence variants (ASVs [120]) and were assigned taxonomy using SILVA (v.132 [121]). The few sequences matching to Archaea and mitochondria as well as singletons were removed. Samples with less than 1000 reads were discarded, and the filtered data table was rarified to 1675 sequences (Table S10)

Amplicon (*16S* rRNA gene) data are available for the developmental stages of 35 sponge species (33 Demospongiae and 2 Homoscleromorpha; Additional file 1: Table S2) [96, 114, 116, 117]. Like the adults, sponge developmental stages associate with a remarkable diversity of bacteria that collectively includes 7794 unique taxa from 40 phyla (Fig. 5A; Additional file 1: Table S2). Any given sponge species associates with between 16 and 272 unique taxa from 4 to 35 phyla. This includes the Proteobacteria (56.3%), Chloroflexi (9.8%), Acidobacteria (7.6%), Actinobacteria (5.2%), Cyanobacteria (4.0%), and Firmicutes (3.6%) (Fig. 5A; Table S3-4). The developmental stages of HMA (14 species) and LMA (21 species) sponges associated with bacterial communities that were comparably similar in total taxa and phylogenetic diversity (*t*-test, observed ASVs: *p* = 0.474; Faith’s: *p* = 0.886; Fig. 5B; Table S5-6). However, the bacterial communities of LMA sponge developmental stages were represented by only a few bacterial taxa while the bacterial communities of HMA sponge developmental stages were more evenly distributed (*t*-test, Mcintosh dominance: *p* = 0.005; Fig. 5B; Table S5).

Two widespread phenomena of sponge-microbe symbioses are that hosts associate with a species-specific and lifestyle-specific microbiome [31, 33]. The developmental stages of HMA and LMA sponges are associated with bacterial communities that are compositionally distinct (PERMANOVA, *p* < 0.001), providing the first demonstration of the HMA-LMA dichotomy in sponge developmental stages. Moreover, the developmental stages of sponges generally, but not always, have a species-specific bacterial community (PERMANOVA, *p* < 0.001; Fig. 5C; Table S7). Pairwise comparison of the 25 sponge species with at least two samples suggest that species specificity occurred in 73.7% (221/300) of the unique combinations (Fig. 5C; Table S7). An inconsistent species-specific signature occurred for 80% (20/25) of the studied sponge species, suggesting that the majority of these species associate with bacterial communities that are compositionally similar to the developmental stages of at least one other sponge. Despite an inconsistent species-specific signature, we compared host phylogeny (using the *18S* rRNA gene; Table S8) and bacterial dendrogram to determine if the symbiont communities covaried with the evolutionary history of the host. Consistent with adult sponges [122], there is topological congruency and, thus, a phylogenetic signal in the symbiont communities of sponge developmental stages (Robinson-Foulds, *p* = 0.025; normalized score 0.904; Fig. 5D; Table S9 [123]).

The diverse bacterial community that the developmental stage of sponges inherit is not composed solely of faithfully transmitted symbionts [44, 113]. Comparisons between adult and larval sponges estimate that larvae share ~45% of their bacterial taxa with the parents, implying that ~55% of the bacterial taxa associated with sponge larvae are acquired horizontally. Moreover, a portion of the vertically transmitted symbionts are microbes that adults acquired from the seawater and subsequently incorporated into the development stages. Patterns of transmission is also inconsistent between siblings, as larvae within a clutch share a small set of identical bacterial taxa (~17%) [89, 113]. Therefore, while adult sponges seed the next generation, there is a degree of stochasticity that has led others to hypothesize that marine sponges exhibit a “leaky” vertical transmission [63, 113]. High taxonomic variability yet functional stability is increasingly common for holobionts that associate with diverse microbial communities [124, 125]. We hypothesize that microbiome function is stable across the sponge life cycle and between generations. This hypothesis remains untested because the meta-omic techniques used on adult sponges have yet to be applied to their developmental stages [30, 126–129].

The vertical transmission of microbial symbionts is a widespread phenomenon among marine sponges. Our understanding of this biological feature stems from taxonomic, geographical, and ecological biases, but, nevertheless, there is a clear spatial coordination during development (Figs. 3 and 4). For a sub-set of these sponge species, we know which symbionts serve as the inoculum for the next generation but not why they are transmitted. The assumption is that there are functional links to the biology and ecology of the dispersing larvae, but data supporting these hypotheses are currently scarce. Microbial symbionts may provide complementary resources to the developing larva once it is no longer sheltered by the adult (Fig. 3P). This may include coping with the dynamic oceanographic environment and seeking out a suitable location to settle and undergo metamorphosis. These are the focal point of the next section: to describe how the microbial communities associated with sponge larvae influence life in the plankton, how these symbiont communities are influenced by the environment, and how they may be key to transition back to the seafloor.

## Life in the plankton

### Pelagic larvae

Marine invertebrates use environmental cues to time reproduction and the release of their eggs, sperm, embryos, and/or larvae to increase fertilization success, dispersal, and food availability [71, 130, 131]. Sponges exhibit reproductive synchrony with annual or bi-annual spawning events, where offspring are released from the excurrent flow of a female sponge in the hours following sunrise or proceeding sunset during particular days or weeks [50, 51, 132–134]. Alternatively, some tropical and subtropical demosponges release small quantities of larvae throughout the year [50, 135]. Upon being released, sponge larvae are between 100 μm and 6 mm in length (i.e., microplankton or mesoplankton), may swim between 0.1 and 1 cm per second, and most have neutral or positive buoyancy [50, 136, 137].

Living in the water column comes with an added mortality risk. The five main sources of mortality for marine invertebrate larvae are temperature, nutritional conditions, dispersal, predation, and metamorphosis [41, 138, 139], but dysbiosis should also be considered [140]. Sponge larvae are non-feeding (lecithotrophic) and spend only a few hours to a few days in the plankton (anchiplanic). Therefore, they are unaffected by external food availability (but not internal nutritional condition) and are less susceptible to being transported off the continental shelf. Mortality due to predation is also likely to be minimal because sponge larvae are briefly in the water column and because they appear to have chemical defenses that make them less palatable [141, 142]. Chemical defenses are symbiont-derived for larvae of the bryozoan *Bugula neritina* [143, 144], and this may also occur in sponges because they are a group rich in secondary metabolites with defensive roles (e.g., [145]). These defenses combined with a short pelagic duration may make sponges favorable for vertical transmission because the microbial cells are less likely to be wasted as a result of larval mortality.

Sponges are released as nearly competent or competent larvae that typically spend minutes to a couple weeks in the water column. Marine invertebrate larvae use a hierarchical suite of ecological signals to locate a suitable site to settle and undergo metamorphosis [50, 134, 146–149]. In the brief hiatus from the benthos, sponge larvae can face substantial local variation in temperature, salinity, and light that influence their dispersal through disruptions in development, locomotion, and behavior [149, 150]. If these ecological variables cause a prolonged larval duration and a delay in metamorphosis, then juvenile fitness will likely be compromised [136, 151]. These same ecological factors may influence animal microbiomes and, in particular, those of marine invertebrate larvae [152, 153]. Larvae from other groups of marine invertebrates horizontally acquire microbes from the seawater in response to variation in abiotic variables [154]. It is estimated that ~55% of the microbial taxa associated with sponge larvae are acquired horizontally [44, 113], but very few experiments have determined whether these horizontal acquisitions also aid in coping with environmental variation.

As human behaviors disproportionately influence global climate, sponge larvae now face an acidified sea with more frequent and prolonged heatwaves that may ultimately compromise their survival, dispersal, and settlement success [155–158]. However, little is known about how these rapid environmental changes affect sponge larval holobionts and their capacity to cope through horizontal acquisitions. Most studies to date have either used *Rhopaloeides odorabile* or *Carteriospongia foliascens*, two sponge species from the Great Barrier Reef. Larvae of these sponges are highly resistant to both acidification and warming, such that an elevated mortality is barely detectable under predicted end-of-the-century conditions [159–162]. These sponge species exhibit taxonomic shifts in their bacterial community by horizontally acquiring microbes in response to temperature as well as crude oil [161, 163]. If and how microbial symbionts influence the larval life stage is unknown, but these pieces of information may be fundamental to predicting the success of sponge larvae in future climatic scenarios.

### Settlement and metamorphosis

As marine invertebrate larvae near competency for metamorphosis, they are guided towards the seafloor by environmental cues and behavioral shifts that are not yet well-understood. Larvae of some species become denser and negatively phototactic, allowing them to sink through the water column, enter the benthic boundary layer, and explore settlement cues [50, 164]. However, this transition cannot occur without the input of specific hormones and/or other signaling compounds that may be acquired exogenously [165, 166] or from symbionts that are both vertically transmitted and metabolically active [45]. One example of the latter is the demosponge *Amphimedon queenslandica*. This sponge requires nitric oxide signaling to stimulate larval settlement, but the host is unable to biosynthesize arginine [167, 168]. Instead, arginine is produced by vertically transmitted bacteria, taken up by the larval cells, and incorporated into the arginine-citrulline loop to produce nitric oxide [169–171]. Metabolic complementation between host and symbiont(s) is widespread [62, 172], but *A. queenslandica* is unique in that this principle extends to a major developmental and life cycle transition [171]. We hypothesize that the symbiont incorporation of signaling that enables larval holobionts to undergo metamorphosis is more widespread among marine invertebrates. Such symbioses may stimulate settlement, but they can only be truly beneficial if the correct benthic substrate is found [173].

The most widespread settlement cue to induce metamorphosis for marine invertebrate larvae are biofilms, complex assemblages of microorganisms that coat most surfaces in the sea [173–177]. Larvae of some marine invertebrates (e.g., the polychaete worm *Hydroides elegans*) must come in contact with a specific bacterium (i.e., P*seudoalteromonas luteoviolacea*) in the biofilm. In this case, *P. luteoviolacea* uses a contractile injection system to induce the settlement of *H. elegans *[174, 178–180]. Our present understanding is that sponge larvae locate a biofilm or conspecific cue to undergo metamorphosis [147, 173, 176]. These responses may be because sponge larvae often have a short planktonic life, and thus, being selective is less advantageous. Alternatively, specific bacterial strains on the seafloor may induce metamorphosis and involve select mechanical triggers [174, 178, 179]. These details remain unresolved in marine sponges.

Metamorphosis involves an extensive reorganization and transdifferentiation of larval cells into adult structures [181–185]. This transition takes hours to days and can be accompanied by a major reconstruction in the associated bacterial community [117, 169]. For example, *Carteriospongia foliascens* has a stable bacterial community throughout this transition, while that of *A. queenslandica* changes drastically due to an influx of environmentally derived bacteria [160, 169]. These associations are short-lived for *A. queenslandica*. Juveniles upregulate genes involved in innate immunity (e.g., scavenger receptors) and host-microbe crosstalk (e.g., ankyrins) that allow for the vertically inherited symbionts to replace these environmental bacteria as the sponge reaches adulthood [169]. The shift away from resident members of the symbiont community would appear to be maladaptive and this interpretation may simply be due to our limited understand of metamorphosis in sponges.

### Post-metamorphosis

For species that did not inherit a full collection of necessary symbionts for life on the benthos, the return to the adult microbiota requires bacterial taxa to be acquired post-metamorphosis via horizontal transmission. The best example of this comes from *Petrosia ficiformis*, an HMA demosponge that does not vertically transmit symbionts to the developmental stages, but associates with archaea and bacteria as adults [73, 103]. The adults are postulated to attract and accumulate microbes from the seawater on its external surface by unknown mechanisms (Fig. 1). Epithelial cells then leave the epithelium to enter the mesohyl (Fig. 1), while folding over itself to form a large extracellular pocket that encloses the symbionts. The resulting mesohyl cell is known as a pocket bacteriocyte (Figs. 1 and 3D) [18]. These are atypical bacteriocytes, in which microbes remain in the extracellular space but still “under control” within the cell pocket. This is in contrast to conventional bacteriocytes in other sponges that host symbionts in true endocytoplamic vesicles (Figs. 1 and 3C, D).

The mode of horizontal transmission described above utilizes “pocketing”—but not endocytosis—of microbes by epithelial cells. Alternatively, symbionts may be acquired from the environment by squeezing between epithelial cells. Sponges have epithelia that are maintained through simple non-tight junctions, allowing a variety of cellular materials (e.g., sponge cells, compounds, and microbes) to enter or exit the mesohyl. Laboratory experiments indicate that microbes may immigrate from the seawater to the mesohyl by squeezing between the epithelial cells [16]. This squeezing is a type of horizontal acquisition that may explain how sponges larvae and juveniles that do not vertically transmit symbiont end up having microbes as the adults. Moreover, “pocketing” may serve as one of the mechanisms used by sponges to exhibit a “leaky” vertical transmission [63, 113].

Our understanding of sponges, their microorganisms, and the transmission of symbionts is deeply rooted in comparative biology (e.g., [35]), and this has allowed for an extensive catalog of microbiome compositions [31], gene inventories [30], and expression profiles [128] to be established in the adults. However, establishing experimental (model) systems often lags when a broad understanding of a given process is first described across multiple species. This is particularly true in marine sponges [8] and their developmental stages, where a single or small set of experimental systems have yet to emerge; the tropical demosponge *A. queenslandica* is currently the frontrunner. In the following section, we outline three groups of techniques that we feel should be established in sponges and their developmental stages to transition research efforts towards characterizing the functional mechanisms of symbiont transmission during reproduction and development.

## Towards the function of developmental symbionts

Experimentally tractable systems in animal-microbe research generally require several desirable properties [8, 186–188]. Establishing a wide array of techniques and accessible genomic data has only been achieved for several animal species, which collectively represent a slim portion of the biological world. Notable advancements have been made on “non-model” animals as holobiont and metaorganism research has popularized, and this is particularly evident in marine sponges [8, 189, 190]. However, there are three primary reproduction and development-focused obstacles that should be overcome for this system to become more experimentally tractable. Specifically, reproduction must be reliably induced in the laboratory, individuals must be amenable to gnotobiotic conditions, and molecular and multi-omic techniques must be developed for host and symbiont.

### Reproduction in the laboratory

Marine invertebrates from most phyla can be reliably induced to reproduce and their offspring can be cultured under laboratory conditions [191, 192]. For example, echinoderms that are collected during their reproductive period can be spawned by an intracoelomic injection of potassium chloride [191–193], and spawning frequency can be manipulated by controlling both temperature and photoperiod [194]. This is also true for the sea anemone *Nematostella vectensis*, which may spawn weekly when provided the proper dietary conditions and photoperiod-temperature combination [195, 196]. Similarly, sponges from a number of genera exhibit predictable and synchronous reproductive events in response to an increase in temperature and a lengthening of the photoperiod (e.g., [76, 197–199]). This reliability has yet to translate to the laboratory. In order to bridge this gap between field and laboratory conditions, we suggest that the thermal regime and photoperiod of spawning events to be characterized for species of interest, and for these conditions to be replicated and manipulated in the laboratory.

### Germ-free sponge larvae

An overarching goal in animal-microbe symbiosis research is to determine the suite of host life-history functions that are impacted by the associated microbial community. An effective way of doing this is by establishing germ-free (axenic) animals, but this is notoriously difficult. For example, it took more than a decade to reliably produce germ-free *Hydra* [200]. Gnotobiotic individuals, where the reduction of the bacterial community is quantified, are more obtainable with the “non-model” animals that disproportionately populate holobiont and metaorganism research. Techniques to generate gnotobiotic marine invertebrate embryos and larvae are limited to the sea anemone *Nematostella vectensis* [201], the sea urchin *Strongylocentrotus purpuratus* [202], and the tunicate *Ciona intestinalis* [203].

The commonalities between these systems are that the developmental stages or early juveniles were incubated in an antibiotic cocktail and subsequently cultured in sterile artificial saltwater, but natural seawater that was fine-filtering and incubated overnight at high temperatures that maintain seawater chemistry can also be used [204]. These protocols have been adopted from the freshwater polyp *Hydra vulgaris* [205] and the zebrafish *Danio rerio* [206], which may collectively serve as a baseline for generating gnotobiotic or germ-free sponge larvae. Preliminary attempts to make the Caribbean sponge *Ectyoplasia ferox* germ-free were promising, as the bacterial community of antibiotically treated larvae—that are contained in a gelatinous sheet rather than disappearing in the open ocean—was significantly reduced but not eliminated [45].

### A molecular toolkit

Much of this synthesis has addressed sponge-symbiont dynamics throughout reproduction and development, but the functional benefits of these partnerships remain largely unknown. Answers to these questions require function-oriented techniques. For example, metagenomic (e.g., [126, 129, 207, 208]) and metatranscriptomic (e.g., [127, 128, 209]) protocols, which are regularly applied to the benthic adults, have yet to be adopted to sponge larvae. The primary bottleneck inhibiting these techniques from being applied to larvae is that they produce insufficient template quantity for sequencing. However, sequencing and nucleic acid extraction sensitivity have advanced substantially in recent years, and technologies maximizing sequencing depth should overcome this barrier, allowing for both symbiont functional potential and activity in the developmental stages to be determined. Establishing these protocols would also afford the opportunity to determine if viruses are vertically transmitted, characterize virome diversity, composition, and structure, and how these relate to the bacterial population [106–109].

The mechanisms underpinning sponge-symbiont interactions during transmission and embryonic development cannot be determined solely by meta-omics, as these techniques only provide snapshots into the biology of sponge holobionts. Meta-omics techniques should be complemented with tools to genetically manipulate both host and symbiont (e.g., CRISPR/Cas9 [210–212]), track host- and symbiont-derived products over time [35, 171], and novel visualization techniques to localize processes of both live and fixed individuals [111, 213]. This information may serve as the basis to use mathematical and computational modeling (e.g., integrated network analysis) to interpret interactions between sponge developmental stages and their symbionts as well as between symbionts [214, 215]. Lastly, this array of techniques can be applied across environmental gradients and over the course of generations to assess hologenomic acclimation and adaptation in vivo [126, 160, 216].

## Developmental symbioses beyond sponges

Coordination between the developing animal host and symbiont community is not unique to marine sponges, as these associations are common among marine and terrestrial animals. The eggs, embryos, and larvae of diverse marine invertebrates (e.g., annelids [217], bivalves [218, 219], bryozoans [143, 220], cnidarians [221, 222], crustaceans [223, 224], echinoderms [152, 225], and gastropods [226]), all associate with microbial symbionts that are interconnected with the holobiont during development [154, 217, 227]. For example, bacteria reside in the external hyaline layer of sea urchin embryos and flood the maturing larval gut lumen once it becomes active [202, 228], where they are able to influence various aspects of aspects of host biology and ecology [154, 225, 227].

Nearly 60 years of research on marine sponges demonstrates that reproduction, development, and symbiont transmission have been deeply rooted for >600 million years [6, 55]. As such, it is increasingly apparent that the internalist view of animal development—where an organism is a mere product of self—will continue to fade as the symbiotic influence comes into focus [229–231]. The ultimate goal in developmental symbiosis of marine sponges, or elsewhere, is not who but why. This—the why—must take center stage in the decades to come because understanding how these interactions and their transmission influence development and the phenotype remain the future of developmental biology.

## Conclusions

In this Review, we have described the general patterns of symbiont transmission in marine sponges during reproduction, development, and metamorphosis. Here, we highlight 5 key points from this Review:

1. Vertical transmission of microbial symbionts through the developmental stages is widespread but not universal, with the majority of species being demosponges from coastal waters of the Mediterranean Sea, Northwestern Atlantic Ocean, and Caribbean Sea.
2. The developmental stages of marine sponges form symbioses with archaea, bacteria, and various eukaryotes (e.g., *Symbiodinum* spp.), with bacteria being the most common and abundant. It remains unknown if viruses are vertically transmitted, but we suspect they are.
3. Symbionts are transmitted to oocytes, embryos, and larvae, with transference beginning at the onset of vitellogenesis and continuing during embryonic cleavage as well as while larvae develop externally.
4. The developmental stages of sponges associate with 10s to 100s of bacterial taxa from upwards of 40 different bacterial phyla and do not always comprise a species-specific microbiome, and species with the HMA and LMA lifestyles have microbiomes distinct in composition. This provides the first demonstration of the HMA-LMA dichotomy in the developmental stages.
5. Little is known about the functional roles of the symbionts associated with the developmental stages and we propose that tools for meta-omic and genetic manipulation of host and symbionts, gnotobiotic culturing of the developmental stages, and crosstalk techniques need to be established.

## Supplementary Information

**Additional file 1: Table S1**. Sponge species with known relations to microorganisms during development and corresponding characteristics. **Table S2**. Sponge developmental stages microbiome sampled used in meta-analysis. **Table S3**. Alpha diversity measures for each sponge developmental stages. **Table S4**. Relative abundance of bacterial phyla for all species of sponge developmental stages. **Table S5**. Summary of statistical tests for alpha diversity between HMA and LMA sponges. **Table S6**. Sponge species with profiled bacterial communities and known as an LMA or HMA. **Table S7**. Summary of PERMANOVA results for compositional comparisons between sponge species and symbiont life-style. **Table S8**. GenBank accession numbers for 18S rRNS sequences used in gene tree as part of phylosymbiosis. **Table S9**. Statistical tables for testing phylosymbiosis across geminate species pairs. **Table S10**. Average alpha rarefaction values for each species based on observed ASVs and phylogenetic diversity [232–267].

**Additional file 2.** The bioinformatic commands used to process and analyze the 16S rRNA sequences that are presented in Figure 6.

## Data Availability

All data used in our *16S* rRNA meta-analysis can be found in Additional file 1: Tables S2 and S8.

## References

[1] Dunn C, Hejnol A, Matus D, Pang K, Browne W, Smith S, Seaver E, Rouse G, Obst M, Edgecombe G (2008). Broad phylogenomic sampling improves resolution of the animal tree of life. Nature.

[2] Van Soest R, Boury-Esnault N, Hooper J, Rützler K, De Voogd N, Alvarez de Glasby B, Hajdu E, Pisera A, Manconi R, Schoenberg C, Janussen D (2018). World Porifera Database. The World Register of Marine Species (WoRMS).

[3] Maldonado M, Ribes M, van Duyl F (2012). Nutrient fluxes through sponges: biology, budgets, and ecological implications. Adv Mar Biol.

[4] Pawlik J, McMurray S (2020). The emerging ecological and biogeochemical importance of sponges on coral reefs. Ann Rev Mar Sci.

[5] Vogel S (1977). Current-induced flow through living sponges in nature. Proc Natl Acad Sci.

[6] Hentschel U, Piel J, Degnan S, Taylor M (2012). Genomic insights into the marine sponge microbiome. Nat Rev Microbiol.

[7] Hentschel U, Usher K, Taylor M (2006). Marine sponges as microbial fermenters. FEMS Microbiol Ecol.

[8] Pita L, Fraune S, Hentschel U (2016). Emerging sponge models of animal-microbe symbioses. Front Microbiol.

[9] Taylor M, Radax R, Steger D, Wagner M (2007). Sponge-associated microorganisms: evolution, ecology, and biotechnological potential. Microbiol Mol Biol Rev.

[10] Thacker R, Freeman C. Sponge-microbe symbioses: recent advances and new directions. In: Becerro M, Uriz M, Maldonado M, Turon X, editors. Advances in Marine Biology, vol. 62. Amsterdam: Elsevier; 2012. p. 57–111. 10.1016/B978-0-12-394283-8.00002-3 22664121

[11] Webster N, Taylor M (2012). Marine sponges and their microbial symbionts: love and other relationships. Environ Microbiol.

[12] Webster N, Thomas T (2016). The sponge hologenome. mBio.

[13] Wilkinson C. Symbiotic interactions between marine sponges and algae. In: Reisser W, editor. Algae and Symbioses: Plants, Animals, Fungi, Viruses, Interactions Explored. Bristol: Lubrecht & Cramer Ltd; 1992.

[14] Azam F, Malfatti F (2007). Microbial structuring of marine ecosystems. Nat Rev Microbiol.

[15] Frost T, Harrison F, Cowden R (1976). Sponge feeding: a review with a discussion of some continuing research. Aspects of Sponge Biology.

[16] Maldonado M, Zhang X, Cao X, Xue L, Cao H, Zhang W (2010). Selective feeding by sponges on pathogenic microbes: a reassessment of potential for abatement of microbial pollution. Mar Ecol Prog Ser.

[17] Schmittmann L, Jahn M, Pita L, Hentschel U. Decoding cellular dialogues between sponges, bacteria, and phages. In: Bosch T, Hadfield M, editors. Cellular Dialogues in the Holobiont. Boca Raton: CRC Press; 2020.

[18] Maldonado M (2007). Intergenerational transmission of symbiotic bacteria in oviparous and viviparous demosponges, with emphasis on intracytoplasmically-compartmented bacterial types. J Mar Biol Assoc UK.

[19] Brien P, Lévi C, Sarà M, Tuzet O, Vacelet J (1973). Traité de Zoologie. Antomie, Systématique, Biologie: Masson et Cie Éditeurs.

[20] Harrison F, De Vos L. Porifera. In: Harrison F, Westfall J, editors. Microscopic Anatomy of Invertebrates, vol. 2. Placozoa, Porifera, Cnidaria, and Ctenophora. Hoboken: John Wiley-Liss; 1991.

[21] de Vos L, Rützler K, Boury-Esnault N, Donadey C, Vacelet J. Atlas of Sponge Morphology. Washington DC: Smithsonian Institution Press; 1991.

[22] Boury-Esnault N, Rützler K. Thesaurus of Sponge Morphology. Smithsonian Contrib Zool. 1997.

[23] Maldonado M. Metazoans: the rise of early animals. In: Vargas P, Zardoya R, editors. The tree of life: evolution and classification of living organisms. Sunderland: Sinauer; 2014. p. 182–205.

[24] Simpson T (1984). The cell biology of sponges.

[25] Stabili L, Licciano M, Longo C, Corriero G, Mercurio M (2008). Evaluation of microbial accumulation capability of the commercial sponge *Spongia officinalis var. adriatica* (Schmidt) (Porifera, Demospongiae). Water Res.

[26] Vacelet J (1975). Etude en microscopie electronique de l’association entre bacteries et spongiaires du genre Verongia (Dictyoceratida). J Microsc Biol Cell.

[27] de Bary A. Die Erscheinung der Symbiose. Strassburg: De Gruyter; 1879.

[28] Oulhen N, Schulz B, Carrier T (2016). English translation of Heinrich Anton de Bary’s 1878 speech, ‘Die Erscheinung der Symbiose’ (‘De la symbiose’). Symbiosis.

[29] Pita L, Rix L, Slaby B, Franke A, Hentschel U (2018). The sponge holobiont in a changing ocean: from microbes to ecosystems. Microbiome.

[30] Robbins S, Song W, Engelberts J, Glasl B, Slaby B, Boyd J (2021). A genomic view of the microbiome of coral reef demosponges. ISME J..

[31] Thomas T, Moitinho-Silva L, Lurgi M, Bjork J, Easson C, Astudillo-García C, Olson J, Erwin P, López-Legentil S, Luter H (2016). Diversity, structure and convergent evolution of the global sponge microbiome. Nat Commun.

[32] Gloeckner V, Wehrl M, Moitinho-Silva L, Gernert C, Schupp P, Pawlik J, Lindquist N, Erpenbeck D, Wörheide G, Hentschel U (2014). The HMA-LMA dichotomy revisited: an electron microscopical survey of 56 sponge species. Biol Bull.

[33] Moitinho-Silva L, Steinert G, Nielsen S, Hardoim C, Wu Y-C, McCormack G, López-Legentil S, Marchant R, Webster N, Thomas T, Hentschel U (2017). Predicting the HMA-LMA status in marine sponges by machine learning. Front Microbiol.

[34] Bayer K, Moitinho-Silva L, Brümmer F, Cannistraci C, Ravasi T, Hentschel U (2014). GeoChip-based insights into the microbial functional gene repertoire of marine sponges (high microbial abundance, low microbial abundance) and seawater. FEMS Microbiol Ecol.

[35] Rix L, Ribes M, Coma R, Jahn M, de Goeij J, van Oevelen D, Escrig S, Meibom A, Hentschel U (2020). Heterotrophy in the earliest gut: a single-cell view of heterotrophic carbon and nitrogen assimilation in sponge-microbe symbioses. ISME J.

[36] Marshall D, Keough M. The evolutionary ecology of offspring size in marine invertebrates. Adv Mar Biol. 2007;53. 10.1016/S0065-2881(07)53001-4 17936135

[37] Vance R (1973). On reproductive strategies in marine benthic invertebrates. Am Nat.

[38] Mousseau T, Fox C (1998). The adaptive significance of maternal effects. Trends Ecol Evol.

[39] Carrier T, Reitzel A, Heyland A. Evolutionary ecology of marine invertebrate larvae. Oxford: Oxford University Press; 2018.

[40] McEdward LR. Ecology of marine invertebrate larvae. Boca Raton: CRC Press; 1995.

[41] Thorson G (1950). Reproductive and larval ecology of marine bottom invertebrates. Biol Rev.

[42] Young C, Sewell M, Rice M, editors. Atlas of Marine Invertebrate Larvae. Cambridge: Academic Press; 2002.

[43] Maldonado M (2009). Embryonic development of verongid demosponges supports the independent acquisition of spongin skeletons as an alternative to the siliceous skeleton of sponges. Biol J Linn Soc.

[44] Schmitt S, Weisz J, Lindquist N, Hentschel U (2007). Vertical transmission of a phylogenetically complex microbial consortium in the viviparous sponge *Ircinia felix*. Appl Environ Microbiol.

[45] Gloeckner V, Lindquist N, Schmitt S, Hentschel U (2013). *Ectyoplasia ferox*, an experimentally tractable model for vertical microbial transmission in marine sponges. Microb Ecol.

[46] Maldonado M, Riesgo A (2009). Reproduction in the phylum Porifera: a synoptic overview. Treballs Societat Catalana Biol.

[47] Riesgo A, Novo M, Sharma P, Peterson M, Maldonado M, Giribet G (2014). Inferring the ancestral sexuality and reproductive condition in sponges (Porifera). Zool Scripta.

[48] Boury-Esnault N, Efremova S, Bezac C, Vacelet J (1999). Reproduction of a hexactinellid sponge: first description of gastrulation by cellular delamination in the Porifera. Invertebr Reprod Dev.

[49] Reiswig H (2004). Hexactinellida after 132 years of study -- what’s new?. Bollettino dei Musei e Degli Istituti Biol dell'Univ Genova.

[50] Maldonado M (2006). The ecology of the sponge larva. Can J Fish Aquat Sci.

[51] Maldonado M, Bergquist P. Phylum Porifera. In: Young C, Sewell M, Rice M, editors. Atlas of Marine Invertebrate Larvae. Cambridge: Academic Press; 2002.

[52] Mileikovsky S (1971). Types of larval development in marine bottom invertebrates, their distribution and ecological significance: a re-evaluation. Mar Biol.

[53] Shanks A (2009). Pelagic larval duration and dispersal distance revisited. Biol Bull.

[54] Strathmann R (1985). Feeding and nonfeeding larval development and life-history evolution in marine invertebrates. Annu Rev Ecol Syst.

[55] Bright M, Bulgheresi S (2010). A complex journey: transmission of microbial symbionts. Nat Rev Microbiol.

[56] Funkhouser L, Bordenstein SR (2013). Mom knows best: the universality of maternal microbial transmission. PLoS Biol.

[57] McFall-Ngai M (2002). Unseen forces: the influence of bacteria on animal development. Dev Biol.

[58] Nyholm S (2020). In the beginning: egg–microbe interactions and consequences for animal hosts. Philos Trans R Soc B.

[59] Nyholm SV, Mcfall-Ngai MJ (2004). The winnowing: establishing the squid-Vibrio symbiosis. Nat Rev Microbiol.

[60] Nyholm S, Stabb E, Ruby E, McFall-Ngai M (2000). Establishment of an animal–bacterial association: recruiting symbiotic vibrios from the environment. Proc Natl Acad Sci.

[61] Nyholm S, McFall-Ngai M (2021). A lasting symbiosis: how the Hawaiian bobtail squid finds and keeps its bioluminescent bacterial partner. Nat Rev Microbiol.

[62] Douglas A (1998). Nutritional interactions in insect-microbial symbioses: aphids and their symbiotic bacteria *Buchnera*. Annu Rev Entomol.

[63] Rodrigues de Oliveira B, Freitas-Silva J, Sanchez-Robinet C, Laport M (2020). Transmission of the sponge microbiome: moving towards a unified model. Environ Microbiol Rep.

[64] Russell S (2019). Transmission mode is associated with environment type and taxa across bacteria-eukaryote symbioses: a systematic review and meta-analysis. FEMS Microbiol Lett.

[65] Dosse G (1939). Bakterien und pilzbefunde sowie pathologische und faülnisvorgänge in meeres und susswasserschwämmen. Z Parasitenkd.

[66] Duboscq O, Tuzet O (1942). Recherches complementaires sur l’ovogenèse, la fécondation et les premiers stades du développement des éponges calcaires. Arch Zool Exp Générale.

[67] Lévi C, Porte A (1962). Ètude au microscope électronique de l'éponge *Oscarella lobularis* Schmidt et de sa larve amphiblastula. Cahiers Biol Mar.

[68] Eckelbarger K (1994). Diversity of metazoan ovaries and vitellogenic mechanisms: implications for life history theory. Proc Biol Soc Washington.

[69] Eckelbarger K, Hodgson A (2021). Invertebrate oogenesis – a review and synthesis: comparative ovarian morphology, accessory cell function and the origins of yolk precursors. Invertebr Reprod Dev.

[70] Koutsouveli V, Cárdenas P, Santodomingo N, Marina A, Morato E, Rapp H, et al. The molecular machinery of gametogenesis in *Geodia* demosponges (Porifera): evolutionary origins of a conserved toolkit across animals. Mol Biol Evol. 2020;37:msaa183. 10.1093/molbev/msaa183 PMC7743902 32929503

[71] Levitan D. The ecology of fertilization in free-spawning invertebrates. In: McEdward L, editor. Ecology of Marine Invertebrate Larvae. Hoboken: CRC Press; 1995.

[72] Fell P, Adiyodi K, Adiyodi R (1989). Porifera. Reproductive Biology of Invertebrates.

[73] Maldonado M, Riesgo A (2009). Gametogenesis, embryogenesis, and larval features of the oviparous sponge *Petrosia ficiformis* (Haplosclerida, Demospongiae). Mar Biol.

[74] Riesgo A, Maldonado M, Durfort M (2007). Dynamics of gametogenesis, embryogenesis, and larval release in a Mediterranean homosclerophorid demosponge. Mar Freshw Res.

[75] Ereskovsky A (2010). The Comparative Embryology of Sponges.

[76] Leys S, Ereskovsky A (2006). Embryogenesis and larval differentiation in sponges. Can J Fish Aquat Sci.

[77] Usher K, Ereskovsky A (2005). Larval development, ultrastructure and metamorphosis in *Chondrilla australiensis* Carter, 1873 (Demospongiae, Chondrosida, Chondrillidae). Invertebr Reprod Dev.

[78] Sarà A, Cerrano C, Sarà M (2002). Viviparous development in the Antarctic sponge *Stylocordyla borealis* Loven, 1868. Polar Biol.

[79] Watanabe Y (1978). The development of two species of Tetilla (Demosponge). Nat Sci Rep Ochanomizu Univ.

[80] Watanabe Y, Masuda Y, Rützler K (1990). Structure of fiber bundles in the egg of *Tetilla japonica* and their possible function in development. *New Perspectives in Sponge Biology*.

[81] Maldonado M. Sponge waste that fuels marine oligotrophic food webs: a re-assessment of its origin and nature. Mar Ecol. 2015;37.

[82] Maldonado M, Cortadellas N, Trillias M, Rützler K (2005). Endosymbiotic yeast maternally transmitted in a marine sponge. Biol Bull.

[83] Schmitt S, Angermeier H, Schiller R, Lindquist N, Hentschel U (2008). Molecular microbial diversity survey of sponge reproductive stages and mechanistic insights into vertical transmission of microbial symbionts. Appl Environ Microbiol.

[84] Riesgo A, Maldonado M (2009). Ultrastructure of oogenesis of two oviparous demosponges: *Axinella damicornis* and *Raspaciona aculeata* (Porifera). Tissue Cell.

[85] Sciscioli M, Liaci L, Lepore E, Gherardi M, Simpson T (1991). Ultrastructural study of the mature egg of the marine sponge *Stelletta grubii* (Porifera, Demospongiae). Mol Reprod Dev.

[86] Lanna E, Klautau M (2010). Oogenesis and spermatogenesis in *Paraleucilla magna* (Porifera, Calcarea). Zoomorphology.

[87] Kaye H (1991). Sexual reproduction in four Caribbean commercial sponges. II. Oogenesis and transfer of bacterial symbionts. Invertebr Reprod Dev.

[88] Sciscioli M, Lepore E, Gherardi M, Liaci L (1994). Transfer of symbiotic bacteria in the mature oocyte of *Geodia cydonium* (Porifera, Demosponsgiae): an ultrastructural study. Cahiers Biol Mar.

[89] Webster N, Taylor M, Behnam F, Lücker S, Rattei T, Whalan S, Horn M, Wagner M (2010). Deep sequencing reveals exceptional diversity and modes of transmission for bacterial sponge symbionts. Environ Microbiol.

[90] Ereskovsky A, Gonobobleva E, Vishnyakov A (2005). Morphological evidence for vertical transmission of symbiotic bacteria in the viviparous sponge *Halisarca dujardini* Johnston (Porifera, Demospongiae, Halisarcida). Mar Biol.

[91] Usher K, Sutton D, Toze S, Kuo J, Fromont J (2005). Inter-generational transmission of microbial symbionts in the marine sponge *Chondrilla australiensis* (Demospongiae). Mar Freshw Res.

[92] Warburton F (1961). Inclusion of parental somatic cells in sponge larvae. Nature.

[93] Levi C, Levi P (1976). Embryogénese de *Chondrosia reniformis* (Nardo), démosponge ovipare, et transmission des bactéries symbiotiques. Annal Sci Naturelles (Zoologie).

[94] de Caralt S, Uriz M, Wijffels R (2007). Vertical transmission and successive location of symbiotic bacteria during embryo development and larva formation in *Corticium candelabrum* (Porifera: Demospongiae). J Mar Biol Assoc UK.

[95] Boury-Esnault N (1976). Ultrastructure de la larve parenchymella *D'hamigera hamigera* (Schmidt) (Démosponge, poecilosclerida) origine des cellules grises. Cahiers Biol Mar.

[96] Busch K, Wurz E, Rapp H, Bayer K, Franke A, Hentschel U (2020). *Chloroflexi* dominate the deep-sea golf ball sponges *Craniella zetlandica* and *Craniella infrequens* throughout different life stages. Front Mar Sci.

[97] Uriz M, Agell G, Blanquer A, Turon X, Casamayor E (2012). Endosymbiotic calcifying bacteria: a new cue to the origin of calcification in Metazoa?. Evolution.

[98] Woollacott R (1993). Structure and swiming behavior of the larva of *Haliclona tubifera* (Porifera: Demospongiae). J Morphol.

[99] Amano S, Hori I (1992). Metamorphosis of calcareous sponges I. Ultrastructure of free-swimming larvae. Invertebr Reprod Dev.

[100] Enticknap J, Kelly M, Peraud O, Hill R (2006). Characterization of a culturable alphaproteobacterial symbiont common to many marine sponges and evidence for vertical transmission via sponge larvae. Appl Environ Microbiol.

[101] Steger D, Ettinger-Epstein P, Whalan S, Hentschel U, de Nys R, Wagner M, Taylor M (2006). Diversity and mode of transmission of ammonia-oxidizing archaea in marine sponges. Environ Microbiol.

[102] Korotkova G, Aisenstadt T (1976). A study of oogenesis of the marine sponge *Haliscarca dujardini*. I. The origin of the oogonia and early stages of oocyte development. Tsitologya.

[103] Lepore E, Sciscioli M, Gherardi M, Laici S (1995). The ultrastructure of the mature oocyte and the nurse cells of the ceractinomorpha *Petrosia ficiformis*. Cahiers Biol Mar.

[104] Schmitt S. Vertical microbial transmission in Caribbean bacteriosponges: Julius-Maximilians-Universität Würzburg; 2007. https://opus.bibliothek.uni-wuerzburg.de/opus4-wuerzburg/frontdoor/deliver/index/docId/2015/file/SchmittPhDthesis2007.pdf.

[105] Gaino E (1980). Indagine ultrastrutturale sugli ovociti maturi dl *Chondrilla nucula* Schmidt (Porifera, Demospongiae). Cahiers Biol Mar.

[106] Jahn M, Arkhipova K, Markert S, Stigloher C, Lachnit T, Pita L, Kupczok A, Ribes M, Stengel S, Rosenstiel P (2019). A phage protein aids bacterial symbionts in eukaryote immune evasion. Cell Host Microbe.

[107] Laffy P, Botté E, Wood-Charlson E, Weynberg K, Rattei T, Webster N (2019). Thermal stress modifies the marine sponge virome. Environ Microbiol Rep.

[108] Laffy P, Wood-Charlson E, Turaev D, Jutz S, Pascelli C, Botté E, Bell S, Peirce T, Weynberg K, van Oppen M (2018). Reef invertebrate viromics: diversity, host specificity and functional capacity. Environ Microbiol.

[109] Pascelli C, Laffy P, Botté E, Kupresanin M, Rattei T, Lurgi M, Ravasi T, Webster N (2020). Viral ecogenomics across the Porifera. Microbiome.

[110] Vacelet J, Gallissian M (1978). Virus-like particles in cells of the sponge *Verongia cavernicola* (Demospongiae, Dictyoceratida) and accompanying tissues changes. J Invertebr Pathol.

[111] Jahn M, Lachnit T, Markert S, Stigloher C, Pita L, Ribes M (2021). Lifestyle of sponge symbiont phages by host prediction and correlative microscopy. ISME J..

[112] Laffy P, Wood-Charlson E, Turaev D, Weynberg K, Botté E, van Oppen M, Webster N, Rattei T (2016). HoloVir: a workflow for investigating the diversity and function of viruses in invertebrate holobionts. Front Microbiol.

[113] Björk J, Díez-Vives C, Astudillo-García C, Archie E, Montoya J (2019). Vertical transmission of sponge microbiota is inconsistent and unfaithful. Nat Ecol Evol.

[114] Moitinho-Silva L, Nielsen S, Amir A, Gonzalez A, Ackermann G, Cerrano C, Astudillo-Garcia C, Easson C, Sipkema D, Liu F (2017). The sponge microbiome project. Gigascience.

[115] Snook R (2005). Sperm in competition: not playing by the numbers. Trends Ecol Evol.

[116] Sacristán-Soriano O, Winkler M, Erwin P, Weisz J, Harriott O, Heussler G, Bauer E, Marsden B, Hill A, Hill M (2019). Ontogeny of symbiont community structure in two carotenoid-rich, viviparous marine sponges: comparison of microbiomes and analysis of culturable pigmented heterotrophic bacteria. Environ Microbiol Rep.

[117] Wu S, Ou H, Liu T, Wang D, Zhao J (2018). Structure and dynamics of microbiomes associated with the marine sponge *Tedania* sp. during its life cycle. FEMS Microbiol Ecol.

[118] Bolyen E, Rideout JR, Dillon MR, Bokulich NA, Abnet C, Al-Ghalith GA, Alexander H, Alm EJ, Arumugam M, Asnicar F (2019). Reproducible, interactive, scalable and extensible microbiome data science using QIIME 2. Nat Biotechnol.

[119] Amir A, McDonald D, Navas-Molina JA, Kopylova E, Morton JT, Xu ZZ, Kightley EP, Thompson LR, Hyde ER, Gonzalez A, Knight R (2017). Deblur rapidly resolves single-nucleotide community sequence patterns. mSystems.

[120] Callahan BJ, McMurdie PJ, Holmes SP (2017). Exact sequence variants should replace operational taxonomic units in marker-gene data analysis. ISME J.

[121] Quast C, Pruesse E, Yilmaz P, Gerken J, Schweer T, Yarza P, Peplies J, Glockner FO (2013). The SILVA ribosomal RNA gene database project: improved data processing and web-based tools. Nucleic Acids Res.

[122] O’Brien PA, Webster NS, Miller DJ, Bourne DG (2019). Host-microbe coevolution: applying evidence from model systems to complex marine invertebrate holobionts. mBio.

[123] Brooks AW, Kohl KD, Brucker RM, van Opstal EJ, Bordenstein SR (2016). Phylosymbiosis: relationships and functional effects of microbial communities across host evolutionary history. PLoS Biol.

[124] Burke B, Steinberg P, Rusch D, Kjelleberg S, Thomas T (2011). Bacterial community assembly based on functional genes rather than species. Proc Natl Acad Sci.

[125] Louca S, Jacques S, Pires A, Leal J, Srivastava D, Parfrey L, Farjalla V, Doebeli M (2016). High taxonomic variability despite stable functional structure across microbial communities. Nat Ecol Evol.

[126] Botté E, Nielsen S, Wahab M, Webster J, Robbins S, Thomas T, Webster N (2019). Changes in the metabolic potential of the sponge microbiome under ocean acidification. Nat Commun.

[127] Moitinho-Silva L, Seridi L, Ryu T, Voolstra CR, Ravasi T, Hentschel U. Revealing microbial functional activities in the Red Sea sponge *Stylissa carteri* by metatranscriptomics. Environ Microbiol. 2014;16:3683–98. 10.1111/1462-2920.12533 24920529

[128] Moitinho-Silva L, Díez-Vives C, Batani G, Esteves A, Jahn M, Thomas T (2017). Integrated metabolism in sponge–microbe symbiosis revealed by genome-centered metatranscriptomics. ISME J.

[129] Slaby B, Hackl T, Horn H, Bayer K, Hentschel U (2017). Metagenomic binning of a marine sponge microbiome reveals unity in defense but metabolic specialization. ISME J.

[130] Morgan S, McEdward L (1995). The timing of larval release. Ecology of Marine Invertebrate Larvae.

[131] Starr M, Himmelman J, Therriault J (1990). Direct coupling of marine invertebrate spawning with phytoplankton blooms. Science.

[132] Amano S (1988). Morning release of larvae controlled by the light in an intertidal sponge, *Callyspongia ramosa*. Biol Bull.

[133] Hoppe W, Reichert M (1987). Predictable annual mass release of gametes by the coral reef sponge *Neofibularia nolitangere* (Porifera: Demospongiae). Mar Biol.

[134] Lindquist N, Bolster R, Laing K (1997). Timing of larval release by two Caribbean demosponges. Mar Ecol Prog Ser.

[135] Kaye H, Reiswig H (1991). Sexual reproduction in four Caribbean commercial sponges. III. Larval behaviour, settlement and metamorphosis. Invertebr Reprod Dev.

[136] Maldonado M, George S, Young C, Vequerizo I (1997). Depth regulation in parenchymella larvae of a demosponge: relative roles of skeletogenesis, biochemical changes and behavior. Mar Ecol Prog Ser.

[137] Maldonado M, Young C (1996). Effects of physical factors on larval behavior, settlement and recruitment of four tropical demosponges. Mar Ecol Prog Ser.

[138] Rumrill S (1990). Natural mortality of marine invertebrate larvae. Ophelia.

[139] Morgan S. Life and death in the plankton: larval mortality and adaptation. In: McEdwards L, editor. Ecology of Marine Invertebrate Larvae. Boca Raton: CRC Press; 1995. p. 279–321.

[140] Carrier T, Macrander J, Reitzel A (2018). A microbial perspective on the life-history evolution of marine invertebrate larvae: if, where, and when to feed. Mar Ecol.

[141] Lindquist N, Hay M (1996). Palatability and chemical defense of marine invertebrate larvae. Ecol Monographs.

[142] Lindquist N (2002). Chemical defense of early life stages of benthic marine invertebrates. J Chem Ecol.

[143] Lopanik N, Lindquist N, Targett N (2004). Potent cytotoxins produced by a microbial symbiont protect host larvae from predation. Oecologia.

[144] Lopanik N, Targett N, Lindquist N (2006). Ontogeny of a symbiont-produced chemical defense in *Bugula neritina* (Bryozoa). Mar Ecol Prog Ser.

[145] Proksch P (1994). Defensive roles for secondary metabolites from marine sponges and sponge-feeding nudibranchs. Toxicon.

[146] Abdul Wahab M, Maldonado M, Luter H, Jones R, Ricardo G (2019). Effects of sediment resuspension on the larval stage of the model sponge *Carteriospongia foliascens*. Sci Total Environ.

[147] Ettinger-Epstein P, Whalan S, Battershill C, de Nys R (2008). A hierarchy of settlement cues influences larval behaviour in a coral reef sponge. Mar Ecol Prog Ser.

[148] Hodin J, Ferner M, Heyland A, Gaylord B, Carrier T, Reitzel A, Heyland A (2018). I feel that! Fluid dynamics and sensory aspects of larval settlement across scales. Evolutionary Ecology of Marine Invertebrate Larvae.

[149] Young C. Behavior and locomotion during the dispersal phase of larval life. In: McEdward L, editor. Ecology of Marine Invertebrate Larvae. Boca Raton: CRC Press; 1995.

[150] Chia F-S, Buckland-Nicks J, Young C. Locomotion of marine invertebrate larvae: a review. Can J Zool. 1984;62.

[151] Pechenik J (2006). Larval experience and latent effects—metamorphosis is not a new beginning. Integr Comp Biol.

[152] Carrier T, Reitzel A (2020). Symbiotic life of echinoderm larvae. Front Ecol Evol.

[153] Kohl K, Carey H (2016). A place for host-microbe symbiosis in the comparative physiologist's toolbox. J Exp Biol.

[154] Carrier T, Reitzel A (2018). Convergent shifts in host-associated microbial communities across environmentally elicited phenotypes. Nat Commun.

[155] Byrne M (2011). Impact of ocean warming and ocean acidification on marine invertebrate life history stages: vulnerabilities and potential for persistence in a changing ocean. Oceanogr Mar Biol: Annu Rev.

[156] Byrne M, Przeslawski R (2013). Multistressor impacts of warming and acidification of the ocean on marine invertebrates’ life histories. Integr Comp Biol.

[157] Hoegh-Guldberg O, Mumby P, Hooten A, Steneck R, Greenfield P, Gomez E, Harvell C, Sale P, Edwards A, Caldeira K (2007). Coral reefs under rapid climate change and ocean acidification. Science.

[158] Oliver E, Donat M, Burrows M, Moore P, Smale D, Alexander L, Benthuysen J, Feng M, Gupta A, Hobday A (2018). Longer and more frequent marine heatwaves over the past century. Nat Commun.

[159] Bennett H, Altenrath C, Woods L, Davy S, Webster N, Bell J (2017). Interactive effects of temperature and pCO2 on sponges: from the cradle to the grave. Glob Chang Biol.

[160] Luter H, Anderson M, Versteegen E, Laffy P, Uthicke S, Bell J (2020). Cross-generational effects of climate change on the microbiome of a photosynthetic sponge. Mol Ecol..

[161] Webster N, Botte E, Soo R, Whalan S (2011). The larval sponge holobiont exhibits high thermal tolerance. Environ Microbiol Rep.

[162] Webster N, Pantile R, Botté E, Abdo D, Andreakis N, Whalan S (2013). A complex life cycle in a warming planet: gene expression in thermally stressed sponges. Mol Ecol.

[163] Luter H, Whalan S, Andreakis N, Wahab M, Botté E, Negri A, Webster N (2019). The effects of crude oil and dispersant on the larval sponge holobiont. mSystems.

[164] Sieber M, Pita L, Weiland-Bräuer N, Dirksen P, Wang J, Mortzfeld B, Franzenburg S, Schmitz R, Baines J, Fraune S (2019). Neutrality in the metaorganism. PLoS Biol.

[165] Bishop C, Brandhorst B (2003). On nitric oxide signaling, metamorphosis, and the evolution of biphasic life cycles. Evol Dev.

[166] Heyland A, Moroz L (2005). Cross-kingdom hormonal signaling: an insight from thyroid hormone functions in marine larvae. J Exp Biol.

[167] Ueda N, Richards G, Degnan B, Kranz A, Adamska M, Croll R, Degnan S (2016). An ancient role for nitric oxide in regulating the animal pelagobenthic life cycle: evidence from a marine sponge. Sci Rep.

[168] Srivastava M, Simakov O, Chapman J, Fahey B, Gauthier M, Mitros T, Richards G, Conaco C, Dacre M, Hellsten U (2010). The *Amphimedon queenslandica* genome and the evolution of animal complexity. Nature.

[169] Fieth R, Gauthier M-E, Bayes J, Green K, Degnan S (2016). Ontogenetic changes in the bacterial symbiont community of the tropical demosponge *Amphimedon queenslandica*: metamorphosis is a new beginning. Front Mar Sci.

[170] Gauthier M-E, Watson J, Degnan S (2016). Draft genomes shed light on the dual bacterial symbiosis that dominates the microbiome of the coral reef sponge *Amphimedon queenslandica*. Front Mar Sci.

[171] Song H, Hewitt O, Degnan S (2021). Arginine biosynthesis by a bacterial symbiont enables nitric oxide production and facilitates larval settlement in the marine-sponge host. Curr Biol.

[172] Dubilier N, Bergin C, Lott C (2008). Symbiotic diversity in marine animals: the art of harnessing chemosynthesis. Nat Rev Microbiol.

[173] Hadfield M, Paul V. Natural chemical cues for settlement and metamorphosis of marine-invertebrate larvae. In: McClintock J, Baker B, editors. Marine Chemical Ecology. Boca Raton: CRC Press; 2001.

[174] Cavalcanti G, Alker A, Delherbe N, Malter K, Shikuma N (2020). The influence of bacteria on animal metamorphosis. Annu Rev Microbiol.

[175] Pawlik J (1992). Chemical ecology of the settlement of benthic marine invertebrates. Oceanogr Mar Biol: Annu Rev.

[176] Whalan S, Webster N (2014). Sponge larval settlement cues: the role of microbial biofilms in a warming ocean. Sci Rep.

[177] Woollacott R, Hadfield M (1996). Induction of metamorphosis in larvae of a sponge. Invertebr Biol.

[178] Shikuma N, Pilhofer M, Weiss G, Hadfield M, Jensen G, Newman D (2014). Marine tubeworm metamorphosis induced by arrays of bacterial phage tail-like structures. Science.

[179] Unabia C, Hadfield M (1999). Role of bacteria in larval settlement and metamorphosis of the polychaete *Hydroides elegans*. Mar Biol.

[180] Ericson C, Eisenstein F, Medeiros J, Malter K, Cavalcanti G, Zeller R, Newman D, Pilhofer M, Shikuma N (2019). A contractile injection system stimulates tubeworm metamorphosis by translocating a proteinaceous effector. Elife.

[181] Amano S, Hori I (1993). Metamorphosis of calcareous sponges II. Cell rearrangement and differentiation in metamorphosis. Invertebr Reprod Dev.

[182] Bergquist P, Green C (1977). An ultrastructural study of settlement and metamorphosis in sponge larvae. Cahiers Biol Mar.

[183] Conaco C, Neveu P, Zhou H, Arcila M, Degnan S, Degnan B, Kosik K (2012). Transcriptome profiling of the demosponge *Amphimedon queenslandica* reveals genome-wide events that accompany major life cycle transitions. BMC Genomics.

[184] Leys S, Kamarul Zaman A, Boury-Esnault N (2016). Three-dimensional fate mapping of larval tissues through metamorphosis in the glass sponge *Oopsacas minuta*. Invertebr Biol.

[185] Nakanishi N, Stoupin D, Degnan S, Degnan B (2015). Sensory flask cells in sponge larvae regulate metamorphosis via calcium signaling. Integr Comp Biol.

[186] Bosch T, Guillemin K, McFall-Ngai M (2019). Evolutionary “experiments” in symbiosis: the study of model animals provides insights into the mechanisms underlying the diversity of host–microbe interactions. BioEssays.

[187] Douglas A (2019). Simple animal models for microbiome research. Nat Rev Microbiol.

[188] Ruby E (2008). Symbiotic conversations are revealed under genetic interrogation. Nat Rev Microbiol.

[189] Bosch T (2020). The model zoologist: how should we think about animals, model animals, and non-model model animals?. Zoology.

[190] Bosch T, McFall-Ngai M (2011). Metaorganisms as the new frontier. Zoology.

[191] Strathmann M. Reproduction and development of marine invertebrates of the northern Pacific coast: data and methods for the study of eggs, embryos, and larvae. Seattle: University of Washington Press; 1987.

[192] Strathmann R. Culturing larvae of marine invertebrates. In: Carroll D, Stricker S, editors. Developmental Biology of the Sea Urchin and Other Marine Invertebrates. Berlin/Heidelberg: Springer; 2014.

[193] Hodin J, Heyland A, Mercier A, Pernet B, Cohen D, Hamel J-F, Allen J, McAlister J, Byrne M, Cisternas P, George S, Foltz K, Hamdoun A (2019). Culturing echinoderm larvae through metamorphosis. Methods in Cell Biology.

[194] Kirchhoff N, Eddy S, Brown N (2010). Out-of-season gamete production in *Strongylocentrotus droebachiensis*: photoperiod and temperature manipulation. Aquaculture.

[195] Darling J, Reitzel AM, Burton P, Mazza M, Ryan JF, Sullivan JC, et al. Rising starlet: the starlet sea anemone, *Nematostella vectensis*. Bioessays. 2005;27:211–21. 10.1002/bies.20181 15666346

[196] Fritzenwanker J, Technau U (2002). Induction of gametogenesis in the basal cnidarian *Nematostella vectensis* (Anthozoa). Dev Genes Evol.

[197] Chaves-Fonnegra A, Maldonado M, Blackwelder P, Lopez J (2015). Asynchronous reproduction and multi-spawning in the coral-excavating sponge *Cliona delitrix*. J Mar Biol Assoc UK.

[198] Witter U, Barthel D, Tendal O (1994). The reproductive cycle of the sponge *Halichondria panicea* Pallas (1766) and its relationship to temperature and salinity. J Exp Mar Biol Ecol.

[199] Wahab M, de Nys R, Webster N, Whalan S (2014). Larval behaviours and their contribution to the distribution of the intertidal coral reef sponge *Carteriospongia foliascens*. PLoS One.

[200] Franzenburg S, Walter J, Kunzel S, Wang J, Baines J, Bosch T, Fraune S (2013). Distinct antimicrobial peptide expression determines host species-specific bacterial associations. Proc Natl Acad Sci U S A.

[201] Domin H, Zurita-Gutiérrez Y, Scotti M, Buttlar J, Hentschel U, Fraune S (2018). Predicted bacterial interactions affect in vivo microbial colonization dynamics in *Nematostella*. Front Microbiol.

[202] Schuh N, Carrier T, Schrankel C, Reitzel A, Heyland A, Rast J. Bacterial exposure mediates developmental plasticity and resistance of lethal Vibrio lentus infection in purple sea urchin (*Strongylocentrotus purpuratus*) larvae. Front Immunol. 2020;10:3014. 10.3389/fimmu.2019.03014 PMC6971090 31993052

[203] Leigh B, Liberti A, Dishaw L. Generation of germ-free *Ciona intestinalis* for studies of gut-microbe interactions. Front Microbiol. 2016;7:2092. 10.3389/fmicb.2016.02092 PMC5186754 28082961

[204] Boje A (2020). Die etablierung einer reproduzierbaren methode, um sterile larven des seeigels *Strongylocentrotus purpuratus* zu produzieren.

[205] Fraune S, Anton-Erxleben F, Augustin R, Franzenburg S, Knop M, Schröder K, Willoweit-Ohl D, Bosch T (2014). Bacteria–bacteria interactions within the microbiota of the ancestral metazoan *Hydra* contribute to fungal resistance. ISME J.

[206] Pham L, Kanther M, Semova I, Rawls J (2008). Methods for generating and colonizing gnotobiotic zebrafish. Nat Protoc.

[207] Rubin-Blum M, Antony C, Sayavedra L, Martínez-Pérez C, Birgel D, Peckmann J, Wu Y-C, Cardenas P, MacDonald I, Marcon Y (2019). Fueled by methane: deep-sea sponges from asphalt seeps gain their nutrition from methane-oxidizing symbionts. ISME J.

[208] Thomas T, Rusch D, DeMaere M, Yung P, Lewis M, Halpern A, Heidelberg K, Egan S, Kjelleberg S (2010). Functional genomic signatures of sponge bacteria reveal unique and shared features of symbiosis. ISME J.

[209] Radax R, Rattei T, Lanzen A, Bayer C, Rapp H, Urich T, et al. Metatranscriptomics of the marine sponge *Geodia barretti*: tackling phylogeny and function of its microbial community. Environ Microbiol. 2012;14:1308–24. 10.1111/j.1462-2920.2012.02714.x 22364353

[210] Ikmi A, McKinney S, Delventhal K, Gibson M (2014). TALEN and CRISPR/Cas9-mediated genome editing in the early-branching metazoan *Nematostella vectensis*. Nat Commun.

[211] Lin C-Y, Su Y-H (2016). Genome editing in sea urchin embryos by using a CRISPR/Cas9 system. Dev Biol.

[212] Neal S, de Jong D, Seaver E (2019). CRISPR/CAS9 mutagenesis of a single r-opsin gene blocks phototaxis in a marine larva. Proc R Soc B.

[213] Geier B, Sogin E, Michellod D, Janda M, Kompauer M, Spengler B, Dubilier N, Liebeke M (2020). Spatial metabolomics of in situ host–microbe interactions at the micrometre scale. Nat Microbiol.

[214] Friedman J, Alm E (2013). Inferring correlation networks from genomic survey data. PLoS Comput Biol.

[215] Kurtz Z, Müller C, Miraldi E, Littman D, Blaser M, Bonneau R (2015). Sparse and compositionally robust inference of microbial ecological networks. PLoS Comput Biol.

[216] Bang C, Dagan T, Deines P, Dubilier N, Duschl W, Fraune S, Hentschel U, Hirt H, Hülter N, Lachnit T (2018). Metaorganisms in extreme environments: do microbes play a role in organismal adaptation?. Zoology.

[217] Vijayan N, Lema KA, Nedved BT, Hadfield MG (2019). Microbiomes of the polychaete *Hydroides elegans* (Polychaeta: Serpulidae) across its life-history stages. Mar Biol.

[218] Salerno J, Macko S, Hallam S, Bright M, Won Y-J, Mckiness Z, Van Dover C (2005). Characterization of symbiont populations in life-history stages of mussels from chemosynthetic environments. Biol Bull.

[219] Sipe A, Wilbur A, Cary S (2000). Bacterial symbiont transmission in the wood-boring shipworm *Bankia setacea* (Bivalvia: Teredinidae). Appl Environ Microbiol.

[220] Woollacott R (1981). Association of bacteria with bryozoan larvae. Mar Biol.

[221] Apprill A, Marlow HQ, Martindale MQ, Rappe MS. Specificity of associations between bacteria and the coral *Pocillopora meandrina* during early development. Appl Environ Microbiol. 2012;78:7467–75. 10.1128/AEM.01232-12 PMC3457095 22904048

[222] Sharp KH, Distel D, Paul VJ. Diversity and dynamics of bacterial communities in early life stages of the Caribbean coral *Porites astreoides*. ISME J. 2012;6:790–801. 10.1038/ismej.2011.144 PMC3309355 22113375

[223] Gil-Turnes M, Hay M, Fenical W (1989). Symbiotic marine bacteria chemically defend crustacean embryos from a pathogenic fungus. Science.

[224] Guri M, Durand L, Cueff-Gauchard V, Zbinden M, Crassous P, Bruce Shillito B, Cambon-Bonavita M-A (2012). Acquisition of epibiotic bacteria along the life cycle of the hydrothermal shrimp *Rimicaris exoculata*. ISME J.

[225] Carrier T, Leigh B, Deaker D, Devens H, Wray G, Bordenstein S, Byrne M, Reitzel A (2021). Microbiome reduction and endosymbiont gain from a switch in sea urchin life-history. Proc Natl Acad Sci.

[226] Klussmann-Kolb A, Brodie G (1999). Internal storage and production of symbiotic bacteria in the reproductive system of a tropical marine gastropod. Mar Biol.

[227] Carrier T, Reitzel A (2019). Bacterial community dynamics during embryonic and larval development of three confamilial echinoids. Mar Ecol Prog Ser.

[228] Cerra A, Byrne M, Hoegh-Guldberg O (1997). Developments of the hyaline layer around the planktonic embryos and larvae of the asteroid Patiriella calcar and the presence of associated bacteria. Invertebr Reprod Dev.

[229] Pradea T (2011). A mixed self: the role of symbiosis in development. Biol Theory.

[230] Gilbert SF, Sapp J, Tauber AI (2012). A symbiotic view of life: we have never been individuals. Q Rev Biol.

[231] Gilbert S (2016). Developmental plasticity and developmental symbiosis: the return of Evo-Devo. Curr Top Dev Biol.

[232] Vacelet J (1999). Planktonic armoured propagules of the excavating sponge *Alectona* (Porifera: Demospongiae) are larvae: evidence from *Alectona wallichii* and *A. mesatlantica* sp. nov. Memoirs Queensland Museum.

[233] Gallissian M, Vacelet J (1976). Ultrastructure de quelques stades de l’ovogenese de spongiares de genre *Verongia* (Dictyoceratida). *ilnnales des Sciences Natllrelles*.

[234] Usher K, Kuo J, Fromont J, Sutton D (2001). Vertical transmission of cyanobacterial symbionts in the marine sponge *Chondrilla australiensis* (Demospongiae). Hydrobiologia.

[235] Lepore E, Sciscioli M, Liaci L, Santarelli G, Gaino G (2000). Sexual reproduction of *Cinachyra tarentina* (Porifera, Demospongiae). Italian J Zool.

[236] Vacelet J, Fiala-Medioni A, Fisher C, Boury-Esnault N (1996). Symbiosis between methane-oxidizing bacteria and a deep-sea carnivorous cladorhizid sponge. Mar Ecol Prog Ser.

[237] Mariani S, Piscitelli M, Uriz M (2001). Temporal and spatial co-occurrence in spawning and larval release of *Cliona viridis* (Porfiera: Hadromerida). J Mar Biol Assoc UK.

[238] Sharp K, Eam B, Faulkner D, Haygood M. Vertical transmission of diverse microbes in the tropical sponge *Corticium sp*. Appl Environ Microbiol. 2007;73:622–9. 10.1128/AEM.01493-06 PMC1796987 17122394

[239] Uriz M, Turon X, Becerro M (2005). Morphology and ultrastructure of the swimming larvae of *Crambe crambe* (Demospongiae, Poecilosclerida). Invertebr Biol.

[240] Koutsouveli V, Taboada S, Moles J, Cristobo J, Rios P, Bertran A, Solà J, Avila C, Riesgo A (2018). Insights into the reproduction of some Antarctic dendroceratid, poecilosclerid, and haplosclerid demosponges. PLoS One.

[241] Bergman O, Haber M, Mayzel B, Anderson M, Shpigel M, Hill R, Ilan M (2011). Marine-based cultivation of *Diacarnus* sponges and the bacterial community composition of wild and maricultured sponges and their larvae. Marine Biotechnol.

[242] Oren M, Steindler L, Ilan M (2005). Transmission, plasticity and the molecular identification of cyanobacterial symbionts in the Red Sea sponge *Diacarnus erythraenus*. Mar Biol.

[243] Sciscioli M, Lepore E (1989). Indagine ultrastructturale sugli ovociti di *Erylus discophorus* (Schmidt) (Porfiera, Tetractinellida). Oebalia.

[244] Gallissian M (1983). Etude ultrastructurale du developpement embryonnaire chez Grontia compressa F. (Porifera, Calcarea). Arch d’Anatomie Microsc.

[245] Gerdts G, Wichels A, Wurst S, Schutt C (2002). Bacterial diversity in the breadcrumb sponge *Halichondria panicea* (Pallas). Papers contributed to the VI International Sponge Conference.

[246] Ereskovsky A, Gonobobleva E (2000). New data on embryonic development of *Halisarca dujardini* Johnston, 1842 (Demospongiae, Halisarcida). Zoosystema.

[247] Ereskovsky A, Boury-Esnault N (2002). Cleavage pattern in *Oscarella* species (Porifera, Demospongiae, Homoscleromorpha): transmission of maternal cells and symbiotic bacteria. J Nat Hist.

[248] Boury-Esnault N, Ereskovsky A, Bezac C, Tokina D (2003). Larval development in the Homoscleromorpha (Porifera, Demospongiae). Invertebr Biol.

[249] Ereskovsky A, Tokina D (2004). Morphology and fine structure of the swimming larvae of *Ircinia oros* (Porifera, Demospongiae, Dictyoceratida). Invertebr Reprod Dev.

[250] Steger D, Ettinger-Epstein P, Whalan S, Hentschel U, de Nys R, Wagner M, Taylor MW (2008). Diversity and mode of transmission of ammonia-oxidizing archaea in marine sponges. Environ Microbiol.

[251] Vishnyakov A, Ereskovsky A (2009). Bacterial symbionts as an additional cytological marker for identification of sponges without a skeleton. Mar Biol.

[252] Gallissian M, Vacelet J (1992). Ultrastructure of the oocyte and embryo of the calcified sponge, *Petrobiona massiliana* (Porifera, Calcarea). Zoomorphology.

[253] Rodrigues de Oliveira B, Lopes I, Canellas A, Muricy G, Dobson A, Laport M. Mot that close to mommy: horizontal transmission majorly influences the microbiota associated with the marine sponge *Plakina cyanorosea*. Microorganisms. 2020:1–24. 10.3390/microorganisms8121978 PMC7764410 33322780

[254] Ruiz C, Villegas-Plazas M, Thomas O, Junca H, Perez T (2020). Specialized microbiome of the cave-dwelling sponge *Plakina kanaky* (Porifera, Homoscleromorpha). FEMS Microbiol Ecol.

[255] Kaye H. Reproduction in West Indian commercial sponges: oogensis, larval develoment and behavior. In: Rutzler K, editor. New Perspectives in Sponge Biology. Washington DC: Smithsonian Institution Press; 1990. p. 161–9.

[256] Lee O, Chui P, Wong Y, Pawlik J, Qian P (2009). Evidence for vertical transmission of bacterial symbionts from adult to embryo in the Caribbean sponge *Svenzea zeai*. Appl Environ Microbiol.

[257] Rutzler K, van Soest RW, Alvarez B (2003). *Svenzea zeai*, a Caribbean reef sponge with a giant larva, and *Scopalina ruetzleri*: a comparative fine-structural approach to classification (Demospongiae, Halichondrida, Dictyonellidae). Invertebr Biol.

[258] Wang J-T, Hirose E, Hsu C-M, Chen Y-Y, Meng P-J, Chen C (2012). A coral-killing sponge, *Terpios hoshinota*, releases larvae harboring cyanobacterial symbionts: an implication of dispersal. Zool Stud.

[259] Sciscioli M, Lepore E, Mastrodonato M, Scalera Liaci L, Gaino E (2002). Ultrastructural study of the mature oocyte of *Tethya aurantium* (Porifera: Demospongiae). Cahiers Biol Mar.

[260] Gaino E, Burlando B, Buffa P, Sará M (1987). Ultrastructural study of the mature egg of *Tethya citrina* Sará & Melone (Porifera, Demospongiae). Gamete Res.

[261] Waterworth S, Jiwaji M, Kalinski J-C, Parker-Nance S, Dorrington R (2017). A place to call home: an analysis of the bacterial communities in two *Tethya rubra* Samaai and Gibbons 2005 populations in Algoa Bay, South Africa. Mar Drugs.

[262] Gaino E, Sara M (1994). An ultrastructural comparative study of the eggs of two species of *Tethya* (Porifera, Demospongiae). Invertebr Reprod Dev.

[263] Collin R, Mobley A, Lopez L, Leys S, Diaz M, Thacker R (2010). Phototactic responses of larvae from the marine sponges *Neopetrosia proxima* and *Xestospongia bocatorensis* (Haplosclerida: Petrosiidae). Invertebr Biol.

[264] Busch K, Wurz E, Rapp H, Bayer K, Franke A, Hentschel U. In: Information NCfB, editor. Chloroflexi dominate the deep-sea golf ball sponges *Craniella zetlandica* and *Craniella infrequens* throughout different life stages; 2020.

[265] Moitinho-Silva L, Nielsen S, Amir A, Gonzalez A, Ackermann G, Cerrano C, Astudillo-Garcia C, Easson C, Sipkema D, Liu F, GitHub (2017). The sponge microbiome project.

[266] Schöttner S, Hoffmann F, Cárdenas P, Rapp H, Boetius A, Ramette A (2013). Relationships between host phylogeny, host type and bacterial community diversity in cold-water coral reef sponges. PLoS One.

[267] Rützler K, Muzik K (1993). *Terpios hoshinota*, a new cyanobacteriosponge threatening Pacific reefs. Scientia Marina.

